# Ricocheting Droplets Moving on Super‐Repellent Surfaces

**DOI:** 10.1002/advs.201901846

**Published:** 2019-09-12

**Authors:** Shuaijun Pan, Rui Guo, Joseph J. Richardson, Joseph D. Berry, Quinn A. Besford, Mattias Björnmalm, Gyeongwon Yun, Ruoxi Wu, Zhixing Lin, Qi‐Zhi Zhong, Jiajing Zhou, Qiang Sun, Jianhua Li, Yanbing Lu, Zhichao Dong, Margaret Katherine Banks, Weijian Xu, Jianhui Jiang, Lei Jiang, Frank Caruso

**Affiliations:** ^1^ State Key Laboratory of Chemo/Biosensing and Chemometrics College of Chemistry and Chemical Engineering Hunan University Changsha 410082 China; ^2^ ARC Centre of Excellence in Convergent Bio‐Nano Science and Technology, and the Department of Chemical Engineering The University of Melbourne Parkville Victoria 3010 Australia; ^3^ Department of Chemical Engineering and the Particulate Fluids Processing Centre The University of Melbourne Parkville Victoria 3010 Australia; ^4^ Department of Materials Department of Bioengineering, and the Institute of Biomedical Engineering Imperial College London London SW7 2AZ UK; ^5^ Zachry Department of Civil Engineering Texas A&M University 3136 TAMU College Station TX 77843‐3136 USA; ^6^ Department of Water Science and Engineering College of Civil Engineering Hunan University Changsha 410082 China; ^7^ CAS Key Laboratory of Bio‐inspired Materials and Interfacial Sciences Technical Institute of Physics and Chemistry Chinese Academy of Sciences Beijing 100190 China

**Keywords:** contact time, droplet bouncing, interfacial phenomena, repellent coatings, surface science

## Abstract

Droplet bouncing on repellent solid surfaces (e.g., the lotus leaf effect) is a common phenomenon that has aroused interest in various fields. However, the scenario of a droplet bouncing off another droplet (either identical or distinct chemical composition) while moving on a solid material (i.e., ricocheting droplets, droplet billiards) is scarcely investigated, despite it having fundamental implications in applications including self‐cleaning, fluid transport, and heat and mass transfer. Here, the dynamics of bouncing collisions between liquid droplets are investigated using a friction‐free platform that ensures ultrahigh locomotion for a wide range of probing liquids. A general prediction on bouncing droplet–droplet contact time is elucidated and bouncing droplet–droplet collision is demonstrated to be an extreme case of droplet bouncing on surfaces. Moreover, the maximum deformation and contact time are highly dependent on the position where the collision occurs (i.e., head‐on or off‐center collisions), which can now be predicted using parameters (i.e., effective velocity, effective diameter) through the concept of an effective interaction region. The results have potential applications in fields ranging from microfluidics to repellent coatings.

## Graphical abstract

**Droplets colliding and ricocheting on super‐repellent surfaces** display dynamics consistent with droplet–surface collisions, but with tunable dynamics between bouncing droplets via control over the angle of incidence. Parameters introduced using the concept of an effective interaction region allow for a universal description and prediction of droplet bouncing dynamics, with implications for wetting characteristics of next‐generation surfaces.

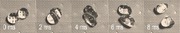

## 1 Introduction

The understanding of droplets bouncing on surfaces (i.e., solid or liquid) has implications for diverse fields and applications including self‐assembly,^1^, ^2^, ^3^ self‐cleaning,^4^ imaging,^5^ anti‐icing,^6^ heat transfer,^7^ fire extinguishing,^8^ fluid transfer,^9^, ^10^ force measurements,^11^ droplet logic,^12^ microfluidics,^13^ sensing,^14^ electronics,^15^ splash dynamics,^16^ wrapping,^17^ and coatings.^18^ Droplet bouncing is expected for collisions with super‐repellent or omniphobic solid surfaces,^19^, ^20^, ^21^, ^22^, ^23^, ^24^, ^25^, ^26^ and the dynamics of single‐droplet bouncing scenarios have been investigated for a variety of surfaces.^27^, ^28^, ^29^, ^30^, ^31^, ^32^, ^33^ Ricocheting droplets, namely, droplet–droplet bouncing, through the collision of two droplets, can occur either in air (i.e., no solid surface involved^34^) or on surfaces (i.e., superhydrophobic surfaces^12^) mediated by thin air films.^33^ However, there are important unanswered questions surrounding the scenario when two droplets collide head‐on or off‐center (e.g., collision outcomes, the scaling of maximum deformation, and contact time)^12^ in regard to whether this falls under the category of droplet–surface collisions or is a unique class of collisions in itself. To date, investigating diverse droplet–droplet collisions has been challenging, largely because a suitable and robust experimental platform for readily manipulating the mobility of probing droplets is lacking.

Herein, we created a super‐repellent surface, which can durably repel a wide range of liquids of interest, by spraying a solution containing fluorosilane and cyanoacrylate onto a deformable copper mesh.^19^ We then used this nearly friction‐free platform to study the bouncing collisions of various liquid droplets and establish predictive parameters for different collision scenarios (i.e., incident collision angles, α). We investigated a spectrum of liquids (i.e., aqueous, organic, and polymer droplets that cover a wide range of physicochemical properties, e.g., surface tension, viscosity, and permittivity) using high‐speed imaging and observed that the collision contact time for all liquids tested (16 liquids) follows the inertia–capillary timescale first encountered by Rayleigh.^35^ We experimentally verified that this scaling holds for droplet bouncing on both super‐repellent solid surfaces and liquid‐droplet surfaces, highlighting that droplet–droplet bouncing is an extreme case of droplet–surface bouncing. Moreover, we found that as the collision becomes more off‐center, the maximum deformation and contact time decreases, but can be scaled to the oscillation period through the use of “effective parameters” that scale the velocity or radius (or both) in relation to the interacting region of each droplet. These results additionally demonstrate the important role of super‐repellent surfaces for studying bouncing dynamics of liquid droplets across a wide range of contact scenarios, and we anticipate that the present study will catalyze investigations into other interfacial repulsion and droplet bouncing phenomena, as well as the engineering of repellent surfaces that display superior liquid mobility.

## 2 Results and Discussion

### 2.1 Super‐Repellent Platform

**Figure 1:**
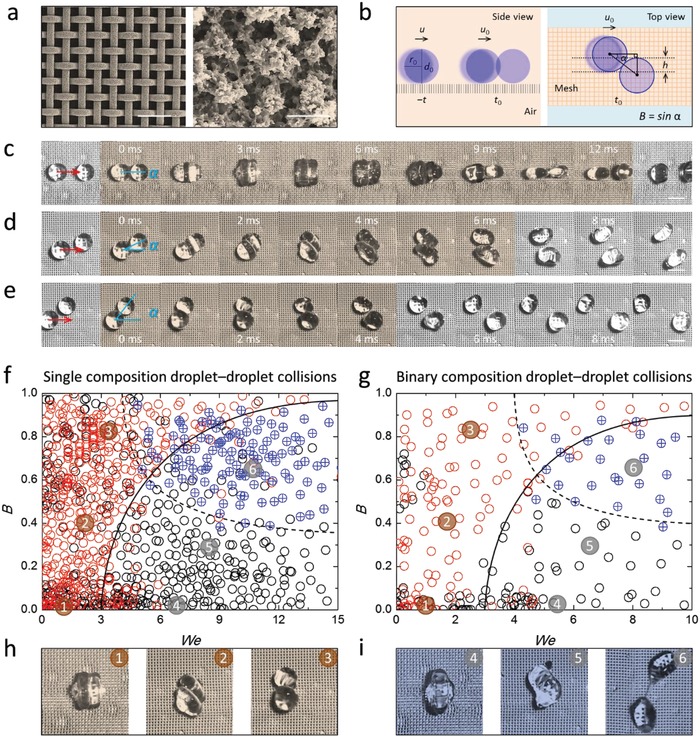
Droplet–droplet collision regimes occurring on a super‐repellent platform. a) Scanning electron microscope images of the super‐repellent surfaces. Scale bars are 500 and 10 µm, respectively. b) Schematics and parameters of the collision between two identical droplets. The moving droplet impacts on the stationary droplet with a speed *u*_0_ at *t* = *t*_0_. The incidence angle is α. *h* is the interaction region along the moving direction upon impact and varies within the range of the droplet diameter *d*_0_. Dimensionless impact parameter *B* is defined as *B* = sinα, varying between 0 and 1 (see detailed *Abbreviations and Definitions*, Supporting Information). c–e) High‐speed image sequences showing the bouncing collision of 1 × 10^−3^ m aqueous sodium dodecyl sulfate (SDS) droplets colliding at different angles α (c, head‐on α ≈ 0°; d, off‐center α ≈ 25°; e, off‐center α ≈ 50°). Scale bars are 2 mm. The brown shaded regions indicate the timescales of two droplets in contact before complete separation. f,g) Collision regimes of single composition droplet–droplet collisions and binary composition droplet–droplet collisions within collision angles of 0°–90° and analytical boundaries (solid and dash) showing bouncing (red), coalescence (black), and stretch separation (blue) events. *B* and *We* are impact parameter and Weber number, respectively. *We* is calculated using the properties of the moving droplet. See detailed *Abbreviations and Definitions* (Supporting Information). The single composition systems (16) include water, 1 m NaCl, 7 m glycerol, 1 mg mL^−1^ PEO_100k_, 0.5 × 10^−3^ m SDS, 1 × 10^−3^ m SDS, 200 × 10^−3^ m SDS, DMF, 5 mg mL^−1^ PMMA_35k_ in DMF, cyclopentanol, *n*‐octanol, *n*‐butanol, ethanol, *n*‐hexadecane, *n*‐dodecane, and *n*‐pentane. The binary composition systems (12) include water–hexadecane, DMF–hexadecane, ethanol–hexadecane, water–ethanol, DMF–ethanol, water–DMF, and the reciprocal collisions. Approximately 30 collisions were studied for each droplet–droplet collision system. See Table S1 (Supporting Information) for additional liquid information. h) Typical bouncing events: head‐on, off‐center, and near‐miss. i) Typical coalescing events: head‐on, off‐center, and near‐miss (i.e., stretching separation).

A super‐repellent surface was first engineered to serve as a platform to facilitate the manipulation of liquid droplets as well as droplet bouncing collisions (**Figure** **1**a). Copper mesh substrates were chosen as they are flexible and ductile, and have the potential to be adapted for different requirements (e.g., bending, indentation). Upon spraying with a solution that contains fluorosilane and cyanoacrylate, a multi‐reentrant hierarchical coating is assembled through a series of synergistic reactions including hydrolysis, condensation, and polymerization (see *Super‐repellent surface* in Methods, Supporting Information).^19^ The resulting super‐repellent platform (consisting of ≈100 µm coarser woven textures and nanoscale finer textures, i.e., 100–1000 nm) displays durable and robust repellence to a diverse range of aqueous and organic liquids (Table S1, Supporting Information), as demonstrated by the free rolling and bouncing of droplets examined on the coated surface (see *Characterization* in Methods, Figure S1, Supporting Information). It is noted that the roll‐off angles of droplets remained constantly negligible (i.e., ≈1°) even after 20 000 cycles of droplet impinging, bouncing, and rolling tests (Figure S1, Supporting Information). By balancing the gravity of the droplet when it starts to roll down the slope (i.e., roll‐off angles are mostly <3°) with the friction between the droplet and the super‐repellent platform, the friction in the system is determined to be within the magnitude of 10^−7^–10^−6^ N for all droplet types (see *Droplet rolling* in Methods, Supporting Information). That is, this negligible friction means that droplets can virtually achieve free locomotion on the super‐repellent platform. The robustness of the surface super‐repellency was investigated through droplet impinging tests by releasing droplets well above the platform. The capillary pressure (i.e., nonwetting pressure) of the super‐repellent coating has been estimated to be about seven times greater than that of the wetting pressures, considering a water droplet is released at a height of 10 cm and impacts on the surface without wetting (see *Droplet bouncing* in Methods, Supporting Information). The durability and robustness as well as the enhanced droplet mobility collectively make this super‐repellent platform suitable for studying diverse droplet collisions including single droplet bouncing and droplet–droplet bouncing (Figure 1b).

### 2.2 Ricocheting Droplets

We examined the collision dynamics of two equal‐sized liquid droplets across a range of surface tensions (15–72 mN m^−1^, typical radius *r*_0_ = 1 mm; Table S1, Supporting Information) occurring on the super‐repellent platform under ambient conditions (i.e., room temperature ≈ 22 °C; relative humidity of ≈40%) via high‐speed imaging. The super‐repellent mesh was bent at the midway point to form a slope relative to a horizontally placed support (Figure S2, Supporting Information). The droplet, once released on the slope, moves along the slope, accelerates and impacts on a stationary droplet preplaced at the flat region along the track of the moving droplet (see *Droplet collision* in Methods, Supporting Information).

Interesting repulsive ricocheting droplets (i.e., droplet–droplet bouncing) occurred on the surface when a rolling droplet collided head‐on with a stationary droplet of the same composition, where both droplets synchronously deformed to the maximal extent allowable by surface tension, followed by retraction and separation without coalescence (Figure 1c; Video S1, Supporting Information). Similarly, when a rolling droplet grazed another droplet (i.e., off‐center collision), they could also bounce off each other before moving away in different directions (Figure 1d,e; Videos S2 and S3, Supporting Information). These droplet–droplet bouncing events, reported in this work, are substantially different from the conventional scenario of a single droplet bouncing on a surface in the gravitational dimension vertically to the surface. To clarify, the droplet bouncing in this work involves two droplets colliding with each other when moving horizontally on a surface in two dimensions.

The defining repulsive feature of the observed droplet‐ricocheting events is that the collision between the two droplets compresses the air between the droplets,^34^ which subsequently drives the droplets away from each other before complete drainage of the air cushion could occur to initiate coalescence. That is, the hydrodynamic pressure within the film is large enough to overcome the attractive intermolecular forces of the colliding droplets.^36^ Our system shares similarities with “walking” droplets on the surface of a liquid^37^, ^38^ and droplet bouncing on the surface of a solid (e.g., wettability‐independent^33^ and super‐repellent bouncing^19^), in that they all involve the maintenance of an “air cushion” with excess pressure at the interface. However, in the “walking” droplets system, the air cushion is maintained by a liquid surface capillary wave,^37^ whereas in wettability‐independent and/or wettability‐dependent droplet bouncing, the solid surface, onto which bouncing occurs, maintains the air cushion by the surface chemistry and/or texture.^19^ In comparison, in our droplet–droplet bouncing system, the air cushion is determined by the droplet–droplet interfacial dynamics mediated by the viscous forces in the free air flow facilitated by the highly porous nature of the super‐repellent surface (Figure 1a).

### 2.3 Collision Regimes

In addition to the above bouncing regimes, droplet–droplet collisions commonly lead to coalescence as well, and the interfacial interactions along with the kinetic forces and viscous forces may play important roles in controlling these collision outcomes. In this work, the probing droplets are all Newtonian liquids with viscosities less than ≈10 mPa s, colliding at relatively small velocities (i.e., <1 m s^−1^). That is, the viscous effect can be largely neglected, while the effect of the kinetic energy and the surface energy should be the leading determining factors. To fully investigate the droplet–droplet collisions occurring on the super‐repellent platform, the collision outcomes were mapped by plotting the Weber number *We* and impact parameter *B* for single liquid droplet–droplet collisions (16 liquids, Figure 1f) and droplet–droplet collisions of liquids of different compositions (12 systems, Figure 1g) (see Figures S3 and S4, Supporting Information). *We* is an important dimensionless parameter for studying fluid dynamics (see other dimensionless parameters in the Supporting Information), which is defined as the ratio of the kinetic energy to the surface energy ($We\;=\rho r_{0}u_{0}^{2}/\gamma \;$, where ρ, *r*_0_, *u*_0_, and γ are the density, radius, impact velocity, and surface tension of the colliding droplet, respectively). The typical collision regimes can be summarized as bouncing (Figure 1h) and coalescence (including stretch separation; Figure 1i and Videos S4–S6, Supporting Information) across the whole range of impact parameters (0 ≤ *B* ≤ 1), which applies for all droplet–droplet collisions within the conditions investigated in the present work (i.e., *We* < 25).

Droplet collisions often lead to permanent coalescence if the impact kinetic energy significantly exceeds the droplet surface energy (i.e., *We* > 6) for low‐impact parameters (i.e., *B* < 0.3). Alternatively, for high‐impact parameters (i.e., *B* > 0.7), collisions can generate stretch separation regimes where the portion of the droplet outside of the interacting region carries enough momentum to overcome the attractive surface forces and can thereby initiate stretch separation.^34^ Permanent coalescence also exists for vanishingly small approach velocities especially for *B* > 0.5, where negligible momentum is transferred upon collision and the drainage of the air layer occurs before the potential droplet–droplet bouncing can be completed. The determination of boundaries between the observed events (i.e., bouncing, coalescence, and stretch separation) can be theoretically computed by comparing the kinetic energy with the surface energy of the effective interaction region *h* (see *Collision boundary* in Methods, Supporting Information). It is noted that the porous super‐repellent platform enables the droplet–droplet collisions to behave similarly to in‐air collisions^34^ provided there is free air flow surrounding the droplets (Figure S5, Supporting Information).

Although the collision regimes can be generally guided by the analytical boundaries, the droplet–droplet collisions experimentally display mixed profiles (i.e., bouncing and coalescing) especially for low impacts (*We* < 5), suggesting that initial conditions may be important for low impact collisions. However, various correlative trends exist between the probability to bounce and the physical properties of the liquid making up the droplet. In general, the coalescence efficiency of droplet–droplet collision decreases as the interactions between the two droplets decrease. Taking the single composition droplet–droplet collisions as an example, collisions between long‐chain alkane droplets (e.g., *n*‐hexadecane droplets) result in a higher probability of droplet–droplet bouncing, which is different to how they behave in droplet–solid surface interactions where they are more likely to spread rather than bounce. Although not the focus of this study, other physical properties (i.e., viscosity, density, surface tension, polarity, and permittivity) have been also comprehensively investigated regarding the coalescence efficiency or the bouncing probability, and the results are summarized in Figure S6 (Supporting Information). In general, low surface tension liquids that are less polar have a higher probability to bounce, whereas polar liquids that have higher surface tensions have a higher probability to coalesce (Figure S6, Supporting Information). Despite this inherent uncertainty in droplet collision dynamics, droplet–droplet bouncing is robust at any given collision condition (i.e., good reproducibility), which makes it feasible to systematically investigate the droplet bouncing dynamics of droplet–droplet collisions.

### 2.4 Head‐on Bouncing Collisions

**Figure 2:**
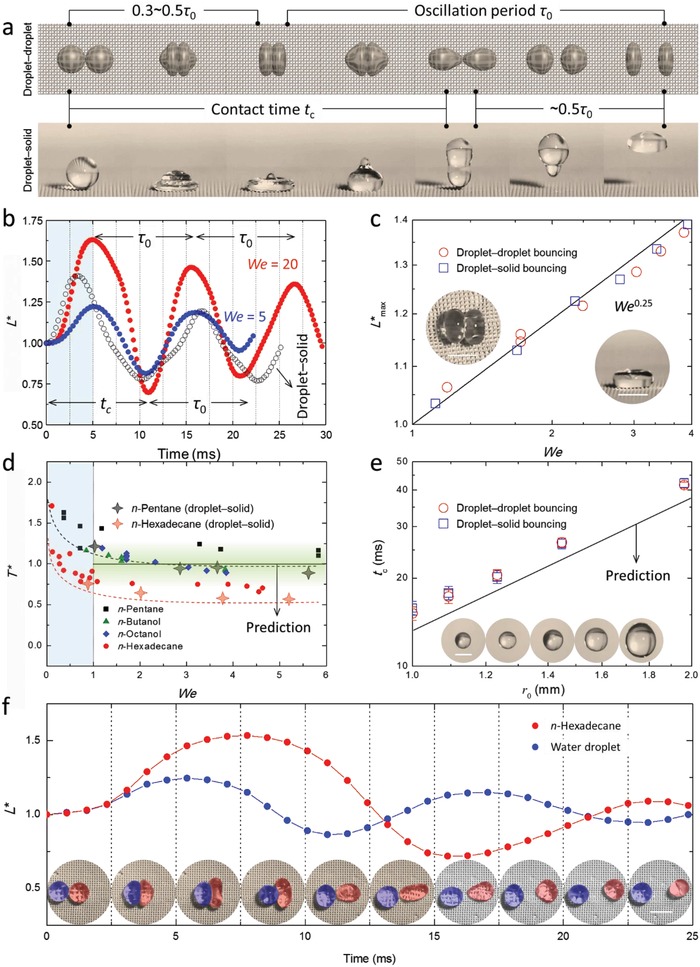
Dynamics of head‐on ricocheting droplets moving on a super‐repellent platform. a) Schematic of head‐on bouncing dynamics between two droplets of the same composition in comparison to single droplet bouncing on a super‐repellent solid surface. During contact, droplets first experience deformation, followed by retraction and oscillation after departure. Each stage represents half of an oscillation period 0.5τ_0_, scaling with the characteristic inertia–capillary timescale $\tau_{0}={\left(\rho d_{0}^{3}/\gamma \right)}^{0.5}\;$, where ρ, *d*_0_, and γ are, respectively, the density, diameter, and surface tension of the liquid. b) Deformation (*l*) of 1 × 10^−3^ m SDS droplets, nondimensionalized (L*) with respect to the initial droplet diameter *d*_0_, L* = *l*/*d*_0_, after the onset of collision for low‐impact (*We* = 5) and high‐impact (*We* = 20) conditions. The droplet–solid bouncing counterpart is shown by open symbols. c) Relationship between maximum deformation (L*_max_) of head‐on bouncing collisions and impact *We* for cyclopentanol droplets, with the solid line representing *We* ^0.25^. The insets show maximum deformation of droplet–droplet bouncing (left) and droplet–solid surface bouncing (right). d) Dependence of contact time (T* is nondimensionalized by the oscillation timescale) on impact *We*. The red and black dashed lines in (d) are to guide the eye. Unified droplet bouncing contact times associated with drops impacting a super‐repellent surface are shown as star symbols for *n*‐pentane (gray) and *n*‐hexadecane (orange). e) Dependence of actual contact time (*t*_c_) on the droplet radius, as demonstrated by head‐on droplet–droplet bouncing (red) and droplet–solid bouncing (blue) collisions of *n*‐octanol, with the solid line representing τ_0_. Error bars in (e) represent standard errors for *n ≥* 5. The insets show the droplet size/volume used for each data point (moving from left to right for data points and drops). f) Droplet–droplet bouncing collision between two droplets of different composition. After collision, the droplets experience individual deformation profiles owing to their differences in surface and viscous forces. The insets are the corresponding collision snapshots during the bouncing collision between water (blue) and oil (i.e., *n*‐hexadecane, red) droplets. The brown shaded regions indicate stages that the two droplets are in contact. Scale bars in (c), (e), and (f) are 2 mm.

We first examined the case where the two colliding droplets are identical (i.e., same composition and equal size). When droplet–droplet bouncing occurs, the colliding droplets act as liquid springs^39^, ^40^ and experience repulsive interfacial dynamics (**Figure** **2**a). The superior mobility of the droplets provided by the super‐repellent platform allows both droplets (i.e., same composition) deform synchronously and follow the general oscillation behavior governed by the surface tension and/or viscosity of the droplets. Figure 2b quantifies the deformation of head‐on colliding droplets (in 1 × 10^−3^ m aqueous SDS) by plotting the dynamic droplet width L* = *l*/*d*_0_ against the droplet oscillation period $\tau_{0}=\sqrt{\frac{\rho d_{0}^{3}}{\gamma }}\;$, where ρ and γ are, respectively, the density and surface tension of the liquid, which represents a general scaling by the characteristic inertia–capillary timescale as first derived by Rayleigh.^35^ It is noted that this inertia–capillary scaling method was primarily used herein and matches well with experimental data in this study considering the negligible effects caused by the viscosity of the probing Newtonian liquids, while other scaling methods^41^, ^42^ (such as viscous scaling and visco‐capillary scaling) are more appropriate when the viscosity effects become significant (Figure S7, Supporting Information). After reaching maximum deformation (i.e., *t* ≈ 5.5 ms in Figure 2b) upon collision, both droplets exhibit oscillator‐like dynamics with the oscillation amplitude dampening with time by liquid viscous forces, and the droplets bounce off each other at *t* = *t*_c_ followed by continuous independent oscillations. The timescale for a colliding droplet reaching maximum deformation is ≈0.5τ_0_ regardless of the initial impact velocity *u*_0_, which has also been proven in the literature^40^ for low *We* collisions (i.e., *We* < 20). By contrast, the *solo* deformation of a bouncing droplet reaches a maximum within a slightly shorter period (≈0.3τ_0_) (see *Maximum deformation* in Methods, Supporting Information). However, both of the maximum deformations (i.e., droplet–solid bouncing, droplet–droplet bouncing) are strictly dependent on the impact kinetic energy. Figure 2c clearly shows the dependence of the maximum deformation on the impact *We*, as demonstrated by collisions between cyclopentanol droplets experiencing head‐on bouncing collisions. As the shape of the colliding droplet results from a balance between the impact force and surface force, L*_max_ necessarily scales as the inverse of the “capillary length”^43^ associated with the colliding droplets ($\lambda^{-1}=\sqrt{\frac{\rho \delta }{\gamma }}\;$, where δ is the acceleration experienced by the colliding droplet). Considering δ scales as $u_{0}^{2}/d_{0}$ and using volume conservation, the maximum deformation (i.e., nondimensionalized) can be deduced as L*_max_ = *We* ^0.25^, which agrees with the observations of the droplet–droplet bouncing collisions and the corresponding droplet–solid surface bouncing using the same super‐repellent surface.^19^, ^43^

As discussed above, the contact time between two head‐on colliding droplets displaying bouncing dynamics is determined by the deformation rate and the retraction rate soon after deformation reaches a maximum, which is strongly related to the scale of the oscillation period τ_0_ (i.e., ≈0.5τ_0_ for each stage). Figure 2d shows the nondimensionalized contact times (T* = *t*_c_/τ_0_) of various liquids (i.e., *n*‐pentane, *n*‐hexadecane, *n*‐octanol, and *n*‐butanol) against the initial impact *We*; the results highlight that all of these droplet–droplet bouncing cases are in agreement with the inertia–capillary timescales rather than viscosity‐based timescales (Figure S7, Supporting Information). Moreover, the current scaling methods work for a wide range of hydrodynamic parameters (Figure S8, Supporting Information) as well as for slightly thicker liquids within the conditions tested (i.e., cyclopentanol; Figure S9, Supporting Information). With consideration to *We*, the colliding droplets bounce off each other with *t*_c_ ≈ τ_0_ when *We* > 1. As for *We* < 1, surface tension forces become dominant, and the dimensionless contact times of the bouncing droplets become larger than one. Additionally, extrapolation of the data reveals that as *We* approaches zero, the contact time doubles, similarly to what is observed in droplet–surface collisions (see *Contact Time* in Methods, Supporting Information).^39^ We find that the trend in contact time of droplet–droplet bouncing is similar to that of single droplet bouncing on super‐repellent surfaces, and that the contact time directly relates to the surface tension of the liquid (Figure S10 and Video S7, Supporting Information). This similarity in bouncing droplets (both droplet–solid and droplet–droplet systems) is also connected to the size dependence of the contact time, as shown in Figure 2e.

We further extended our study to the droplet–droplet bouncing collisions of two droplets with different compositions (binary system) (Figure 1g; Figure S4, Supporting Information). The collision regimes simply follow that of droplet–droplet collisions of single liquid systems (i.e., three regimes: coalescence, bouncing, and separation). Despite the physical differences between the two droplets in the binary system, their bouncing collisions still behave like two individual oscillators in terms of deformation and contact time (Figure 2f; Video S8, Supporting Information). As the extent of droplet deformation is a function of the relative impacting kinetic energy over the droplet surface energy, an oil droplet that has a lower surface tension deforms to a greater extent than its water counterpart (L* ≈ 1.5 compared to ≈1.2, respectively). Additionally, the apparent contact time between two colliding droplets in the binary system falls within their individual oscillation periods (i.e., τ_water_ < *t*_c_ < τ_oil_) (see *Oscillator* in Methods, Supporting Information). When the oscillation periods are scaled with the relevant liquid parameters (density, initial diameter, and surface tension), the dimensionless oscillation period for each droplet becomes ≈1.0, in agreement with the characteristic inertia–capillary timescale. This is an important insight for common droplet–droplet interactions in industrial applications where the compositions are not necessarily identical. It is also noted that the nonsynchronous deformation of the droplets involved in the binary composition droplet–droplet bouncing collisions also can be partially attributed to the slight difference in the interactions between the liquids and the solid platform as well as the difference in the viscous dissipations. However, in the current study (of reasonably small impact kinetic energy), the effects of surface friction and liquid viscosity can be virtually neglected. All of these factors account for the applicability of the above contact timescaling in regard to the bouncing collisions between two distinct droplets.

### 2.5 Off‐Center Bouncing Collisions

**Figure 3:**
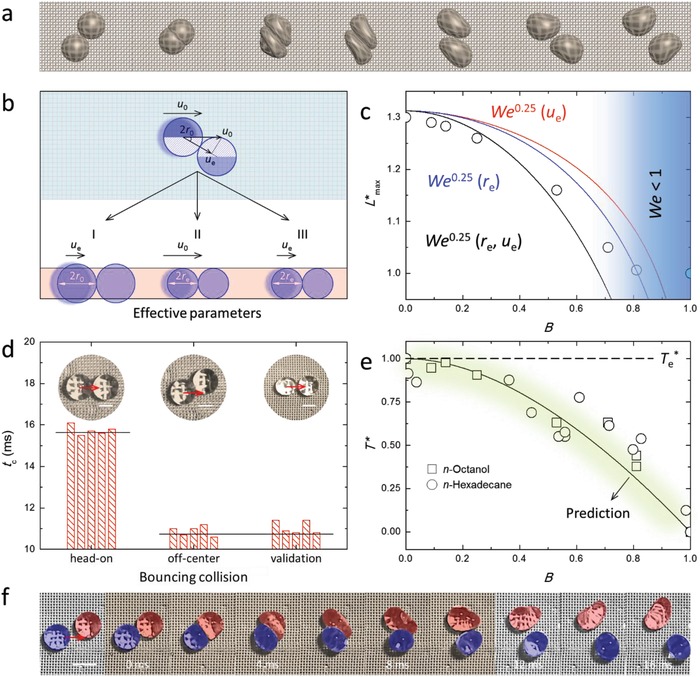
Dynamics of off‐center ricocheting droplets moving on a super‐repellent platform. a) Schematic of off‐center bouncing dynamics between two droplets of the same composition. b) Schematic of the three equivalent head‐on scenarios for off‐center collision considering the effective parameters. Case I, effective impact velocity *u*_e_ (i.e., *u*_e_ = *u*_0_cosα). Case II, effective interaction diameter *d*_e_ (i.e., $d_{e}=d_{0}\;{\left(1-B\right)}^{\frac{2}{3}}{\left(1\;+\;2B\right)}^{\frac{1}{3}}$). Case III, the combination of Case I and Case II. See *Effective parameters* in Methods (Supporting Information) for more details. c) Maximum deformation of bouncing *n*‐octanol collisions versus the impact parameter. Solid lines are predicted using the corresponding effective parameter for *We* ^0.25^ (red, effective velocity *u*_e_; blue, effective radius *r*_e_; black, effective radius and effective velocity *r*_e_, *u*_e_). d) Experimental validation of the “effective collision” assumption using *n*‐octanol droplets. Insets indicate the onset of the collisions. Scale bars are 1 mm. e) Contact time for off‐center bouncing collisions for *n*‐hexadecane (circle) and *n*‐octanol (square). The black curve and the dashed line are the predicted contact times using the initial head‐on collision (*h* = *d*_0_) as the reference or using the effective counterpart (*h* = *d*_e_) as the reference. f) Off‐center bouncing collision between two droplets of different composition (i.e., a water droplet (blue) impacts on a *n*‐hexadecane droplet (red) of identical size, *B* ≈ 0.47). The timescale is indicated by the brown region. Scale bar is 2 mm.

The dynamics of off‐center bouncing collisions are more complex than for head‐on bouncing collisions, and the droplet deformation significantly deviates from the conventional “pancake” shape seen in head‐on collisions (**Figure** **3**a). Therefore, the prediction of off‐center bouncing dynamics (i.e., L*_max_, T*) is challenging, however the rational prediction of off‐center collisions is needed for practical application in the industrial, engineering, and scientific realms. For off‐center droplet–droplet bouncing, an important parameter is the region *h*, where the initial interaction upon impact mainly occurs along the direction of droplet locomotion. For identical droplets (i.e., same *r*_0_), the interaction region can be computed according to the placement of the droplets at the onset of the collision (i.e., the overlap of the two droplets), that is, *h* = 2*r*_0_(1 − *B*). For head‐on collisions, *h* equals the diameter of the droplet 2*r*_0_. That is, the effective momentum decreases as the point of collision deviates from the droplet center (i.e., *B* > 0). Considering that the timescale of droplet–droplet bouncing is transient (largely <20 ms for millimeter‐sized droplets), this effective interaction region *h* might dominate the virtual dynamics.

To address this, we propose effective parameters deduced by considering the collision angle for off‐center collisions (i.e., effective velocity *u*_e_ = *u*_0_cosα, which is the velocity vector along the line connecting the two centers of mass of the colliding droplets, and effective radius $r_{e}=r_{0}\;{\left(1-B\right)}^{\frac{2}{3}}{\left(1\;+\;2B\right)}^{\frac{1}{3}}$, which is derived using volume conservation of the interacting region^34^) (see *Effective parameters* in Methods, Supporting Information). Three scenarios can be considered in regard to an off‐center collision as shown in Figure 3b. That is, an off‐center collision between two droplets (i.e., *r*_0_, *u*_0_, *B >* 0) might have three head‐on equivalents (i.e., I, II, III; *B* = 0). Scenario I is a head‐on collision between two droplets with actual sizes at an effective velocity (i.e., *r*_0_, *u*_e_). Scenarios II and III are the head‐on collisions between two droplets with reduced (effective) sizes *r*_e_ either at the original velocity *u*_0_ or the effective velocity *u*_e_, respectively. Based on these assumptions, Figure 3c compares three prediction scenarios (i.e., considering solely the effective velocity or radius, or considering the two variables simultaneously) using effective Weber numbers for bouncing collisions of *n*‐octanol droplets. By considering the interaction regions as two equivalent smaller spherical droplets and using the effective impact velocity, the predictions agree with the experimental results for small collision angles (i.e., *B* < 0.5). It is noted that the above scenarios do not hold for large grazing angles (i.e., *B* > 0.8) because the droplets do not significantly deform as the effective Weber numbers approach zero and surface tension dominates (potentially due to the negligible hydrodynamic interactions).

Similarly, to predict the contact time of off‐center droplet–droplet bouncing collisions, effective parameters can be considered as well. As demonstrated above, the contact time of the bouncing droplet is independent of impact velocity; however, it directly relates to the nature of droplet oscillation. Thus, we only considered the “effective volume”^29^ or “effective radius” (i.e., the equivalent volume or radius of the interaction region upon contact) for evaluating and validating the contact time of off‐center bouncing collisions (see *Effective parameters* in Methods, Supporting Information). Figure 3d compares the contact times of three bouncing collisions—a reference head‐on bouncing collision (*t*_c_ ≈ *t*_0_, ≈15.7 ms), an off‐center bouncing collision (*t*_c_ ≈ 10.9 ms) between two droplets (*r*_0_, *B* ≈ 0.5), as well as the experimentally equivalent “effective” case (i.e., head‐on bouncing collision between two smaller counterparts, *r*_e_ ≈ 0.8*r*_0_; *t*_c_ ≈ 11.0 ms). Moreover, based on the effective dimension of the droplets involved in off‐center bouncing collisions, the contact time can be scaled as τ_e_ = τ_0_ (1 − *B*)(1  +  2*B*)^0.5^ (see *Contact time* in Methods, Supporting Information). The scaling gives a predicted value of 11.0 ms for the above off‐center bouncing collision, which is also in strong agreement with the experimental data. Figure 3e further validates that the “effective collision” assumption matches the experimental results, as demonstrated by various droplet–droplet bouncing collisions at different collision angles (Figure S11, Supporting Information) and is in agreement with the universal scaling of Rayleigh's oscillation (i.e., inertia–capillary timescale). When normalized with the effective oscillation periods τ_e_, the contact times of all off‐center bouncing collisions (i.e., 0 ≤ *B* ≤ 1) can potentially fall into *T*_e_* = 1, making it feasible to compare them with all other bouncing collisions regardless of the bouncing conditions.

Similarly, the concept of “effective” collisions also works for the prediction of contact times between two droplets with different compositions (binary system) occurring for off‐center droplet–droplet bouncing collisions (Figure 3f; Video S9, Supporting Information). The contact time of a water–hexadecane off‐center bouncing collision (*B* ≈ 0.47) has been experimentally measured with a value of 9.5 ms. The value is estimated to be in the range of 8–11 ms as predicted using the above effective scaling method τ_e_ using the characteristic timescales of water (τ_0_ = 10.5 ms) and oil (τ_0_ = 15.0 ms) droplets, respectively. As revealed in Figure 2f, it is also suggested that the determination of the contact time of the binary composition droplet–droplet systems, including head‐on and off‐center bouncing collisions, needs to take into account both dynamic behaviors of the probing droplets.

### 2.6 Universal Droplet Bouncing

**Figure 4:**
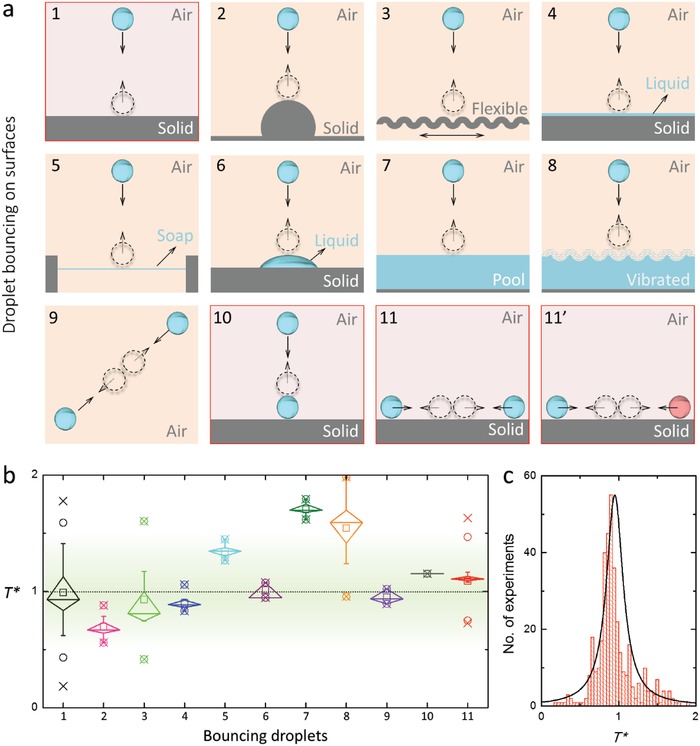
Droplet bouncing scenarios and nondimensionalized contact times. a) Illustrations of droplet bouncing scenarios examined in the present and literature studies. Impacting droplets and liquids are illustrated in blue; rebounding droplets are presented by dashed circles; and the solid and dashed arrows indicate the movements of the impacting and rebounding droplets, respectively. Droplet bouncing: 1, on a solid planar surface; 2, on a curved solid surface; 3, on a flexible solid surface; 4, on a thin liquid film; 5, on a soap film; 6, on a sessile droplet adhered to a solid surface; 7, on a liquid pool; 8, on a vibrating liquid pool; 9, between two droplets in air; 10, vertically onto another droplet residing on a super‐repellent surface; and 11, between two droplets of identical (11) or different (11′) composition on a super‐repellent surface. b) Corresponding box charts of nondimensionalized contact times of bouncing droplets illustrated in 1–11′ (axis label 11 comprises data for scenarios illustrated in 11 and 11′). c) Histogram of the nondimensionalized contact times reported in the literature and the present study. Note, experimental data were collected herein for scenarios 1, 10, 11, and 11′ and combined with data from the literature (scenarios 1–10) in (b) and (c) (see refs. [S3, S5, S6, S9, S14–S72], Supporting Information); thus, data for scenarios 11 and 11′ are unique to the present study. See Tables S2 and S3 (Supporting Information) for more details.

All of the cases of droplet–droplet bouncing measured in this study (28 cases) can be described using Rayleigh's inertia–capillary scaling method. Next, we determined the oscillation timescale of other droplet bouncing scenarios to determine whether droplet–droplet bouncing is unique or a subset of a universal description of droplet–solid surface bouncing. Specifically, we experimentally validated horizontal droplet–droplet collisions, vertical droplet–droplet collisions, and single droplets bouncing on a super‐repellent surface (Figure S12, Supporting Information), and we analyzed and transformed the relevant data from the literature on droplet bouncing on wetting solid^33^ or curved surfaces,^44^ droplet bouncing on thin liquid films^28^ or deep liquid baths,^45^ droplet bouncing on a sessile droplet,^46^ and droplet–droplet bouncing collisions occurring in air^47^ (**Figure** **4**a). Importantly, we find that all 16 liquids examined in the present study give T* values in the range of 0.8–1.2, and the 63 literature scenarios analyzed largely give T* = *t*_c_/$\sqrt{\frac{\rho d_{e}^{3}}{\gamma }}$ values in the range of 0.8–1.4 (i.e., with averages of ≈1.0 ± 0.3) (Figure 4b,c; Tables S2 and S3, Supporting Information), with the higher deviation likely related to the need for interpreting images and lack of raw data provided in the literature examples. These results, collectively, strongly suggest that the inertia–capillary scaling^35^ is universal for droplet bouncing scenarios mediated by a thin layer of air, that is, air lubrication^33^, ^36^ (note that the liquids involved are mostly Newtonian liquids, and the viscous effect is negligible).

## 3 Conclusion

Collisions between droplets moving on a super‐repellent platform may result in bouncing, coalescence, or stretch separation. Ricocheting droplets, of either identical or distinct chemical compositions, behave similarly to drops bouncing on soft and solid surfaces. The maximum deformation of the drops and contact time between drops are highly dependent on the position where the collision occurs (i.e., head‐on or off‐center collisions); both parameters decrease with increasing angle of incidence. Parameters have been introduced using the concept of effective interaction region, allowing for accurate prediction of droplet bouncing dynamics and providing a simple and universal way to compare other droplet bouncing scenarios (e.g., droplet bouncing on diverse surfaces). Droplet collision dynamics are related to the all‐round evaluation of super‐repellent surfaces, with importance for various practical applications such as selective momentum transfer and mass transfer for droplet–droplet collisions between liquids of identical or different compositions (e.g., dyed systems). The dynamics of droplet collisions allow for the understanding of different collision regimes as well as the rational design of next‐generation surfaces.^48^, ^49^, ^50^ Finally, the framework developed herein could serve as a platform^51^ for future research, both theoretical and applied.

## Conflict of Interest

The authors declare no conflict of interest.

## Supporting information

Supplementary

Supplementary

Supplementary

Supplementary

Supplementary

Supplementary

Supplementary

Supplementary

Supplementary

Supplementary
