# Supplementary material for: Ricocheting Droplets Moving on Super‐Repellent Surfaces
Source: Adv Sci (Weinh). 2019 Sep 12;6(21):1901846. doi: 10.1002/advs.201901846 (PMC6839626; doi:10.1002/advs.201901846)
Supplement: Supplementary file 1 — Supplementary [file ADVS-6-1901846-s002.pdf]

Copyright WILEY-VCH Verlag GmbH & Co. KGaA, 69469 Weinheim, Germany, 2019.

ADVANCED SCIENCE
Open Access

Supporting Information

for *Adv. Sci.,* DOI: 10.1002/advs.201901846

Ricocheting Droplets Moving on Super-Repellent Surfaces

Shuaijun Pan, Rui Guo, Joseph J. Richardson, Joseph D. Berry, Quinn A. Besford, Mattias Björnmalm, Gyeongwon Yun, Ruoxi Wu, Zhixing Lin, Qi-Zhi Zhong, Jiajing Zhou, Qiang Sun, Jianhua Li, Yanbing Lu, Zhichao Dong, Margaret Katherine Banks, Weijian Xu, Jianhui Jiang, Lei Jiang, and Frank Caruso**

Copyright WILEY-VCH Verlag GmbH & Co. KGaA, 69469 Weinheim, Germany, 2018.

Supporting Information

Ricocheting droplets moving on super-repellent surfaces

Shuaijun Pan, Rui Guo, Joseph J. Richardson, Joseph D. Berry, Quinn A. Besford, Mattias Björnmalm, Gyeongwon Yun, Ruoxi Wu, Zhixing Lin, Qi-Zhi Zhong, Jiajing Zhou, Qiang Sun, Jianhua Li, Yanbing Lu, Zhichao Dong, M. Katherine Banks, Weijian Xu, Jianhui Jiang, Lei Jiang, and Frank Caruso**

Dr. S. Pan, R. Guo, Prof. Y. Lu, Prof. W. Xu, Prof. J. Jiang
State Key Laboratory of Chemo/Biosensing and Chemometrics, College of Chemistry and Chemical Engineering, Hunan University, Changsha 410082, China
E-mail: pansj@hnu.edu.cn

Dr. S. Pan, R. Guo, Dr. J. J. Richardson, Dr. Q. A. Besford, Dr. M. Björnmalm, Dr. G. Yun, Z. Lin, Q.-Z. Zhong, Dr. J. Zhou, Dr. J. Li, Prof. F. Caruso
ARC Centre of Excellence in Convergent Bio-Nano Science and Technology, and the Department of Chemical Engineering, The University of Melbourne, Parkville, Victoria 3010, Australia
E-mail: fcaruso@unimelb.edu.au

Dr. J. D. Berry, Dr. Q. Sun
Department of Chemical Engineering and the Particulate Fluids Processing Centre, The University of Melbourne, Parkville, Victoria 3010, Australia

Dr. M. Björnmalm
Department of Materials, Department of Bioengineering, and the Institute of Biomedical Engineering, Imperial College London, London SW7 2AZ, UK

Prof. R. Wu, Prof. M. K. Banks
Zachry Department of Civil Engineering, Texas A&M University, 3136 TAMU, College Station, TX 77843–3136, USA

Prof. R. Wu
Department of Water Engineering and Science, College of Civil Engineering, Hunan University, Changsha 410082, China

Prof. Z. Dong, Prof. L. Jiang
CAS Key Laboratory of Bio-inspired Materials and Interfacial Sciences, Technical Institute of Physics and Chemistry, Chinese Academy of Sciences, Beijing 100190, China

Video Captions

Video S1. 1 mM SDS droplets head-on bouncing collision (*We* = 20.0, *B* = 0.01). The video was recorded at 5,155 frames per second and plays at 30 frames per second.

Video S2. 1 mM SDS droplets off-center bouncing collision (*We* = 8.9, *B* = 0.43). The video was recorded at 5,155 frames per second and plays at 30 frames per second.

Video S3. 1 mM SDS droplets off-center bouncing collision (*We* = 7.4, *B* = 0.76). The video was recorded at 5,155 frames per second and plays at 30 frames per second.

Video S4. 1 mM SDS droplets head-on coalescence (*We* = 17.5, *B* = 0.01). The video was recorded at 5,155 frames per second and plays at 30 frames per second.

Video S5. 1 mM SDS droplets off-center coalescence (*We* = 3.9, *B* = 0.75). The video was recorded at 5,155 frames per second and plays at 30 frames per second.

Video S6. 1 mM SDS droplets off-center stretch separation (*We* = 9.0, *B* = 0.77). The video was recorded at 5,155 frames per second and plays at 30 frames per second.

Video S7. 1 mM SDS droplet bouncing on super-repellent surface (*We* = 3.1). The video was recorded at 3,900 frames per second and plays at 30 frames per second.

Video S8. Binary liquid droplet–droplet system head-on bouncing (water moving towards hexadecane; *We* = 0.4, *B* = 0.02). The video was recorded at 5,155 frames per second and plays at 30 frames per second.

Video S9. Binary liquid droplet–droplet system off-center bouncing (water moving towards hexadecane; *We* = 3.1, *B* = 0.47). The video was recorded at 5,155 frames per second and plays at 30 frames per second.

Abbreviations and Definitions

α	Incident collision angle between the direction of the moving droplet and the normal through both droplet centers when collision occurs, or the angle between the moving directions of the two droplets: $\alpha = \arcsin B = \arcsin \frac{\chi}{2r_0}$
B	Impact parameter: $B = \sin\alpha$ or $B = \frac{\chi}{2r_0}$
χ	Projected distance between droplet centers in the plane perpendicular to the direction of the moving droplet: $\chi = 2r_0 B$
γ	Surface tension of the liquid
r_0	Initial droplet radius: $r_0 = \frac{d_0}{2}$
d_0	Initial droplet diameter: $d_0 = 2r_0$
μ	Viscosity of liquid
We	Weber number: $We = \frac{\rho r_0 u_0^2}{\gamma}$, the ratio of the kinetic energy to the surface energy
Oh	Ohnesorge number: $Oh = \frac{\mu}{\sqrt{\rho r_0 \gamma}}$, the ratio of the viscous forces to the inertial and surface forces
Re	Reynolds number: $\mathrm{Re} = \frac{\rho r_0 u_0}{\mu}$, the ratio of the inertial to the viscous forces
Ca	Capillary number: $Ca = \frac{\mu u_0}{\gamma}$, the ratio of viscous forces to the surface tension
La	Laplace number: $La = \frac{\rho r_0 \gamma}{\mu}$, the ratio of the surface tension to the viscous dissipation
Ga	Galilei number: $Ga = \frac{g r_0^3}{\mu^2}$, the ratio of the gravitational to the viscous forces
Bo	Bond number: $Bo = \frac{\rho g r_0^3}{\gamma}$, the ratio of capillary to the gravitational forces

τ_0	Period of an oscillating droplet: $\tau_0 = \sqrt{\frac{\rho d_0^3}{\gamma}}$
τ_{water}	Oscillation period of a water droplet
τ_{oil}	Oscillation period of an oil droplet
t_{c}	Contact time of the bouncing droplet with the solid or liquid surfaces
l	Deformation: diameter of the droplet perpendicular to the normal through both droplet centers when collision occurs
l_{max}	Maximal deformation during droplet bouncing
L^*	Dimensionless deformation with respect to the initial droplet diameter: $L^* = \frac{l}{d_0}$
L^*_{max}	Maximal dimensionless deformation
T^*	Dimensionless contact time with respect to the droplet oscillation period: $T^* = \frac{t_{\text{c}}}{\tau_0}$
ρ	Density of the droplet
N	Number of measurements
λ	Capillary length of the liquid: $\lambda = \sqrt{\frac{\gamma}{\rho\delta}}$
δ	Acceleration experienced by the colliding droplet: $\delta \propto \frac{u_0^2}{d_0}$
u_0	Initial velocity of the colliding droplet
u_{e}	Effective velocity of the droplet initiating the off-center collision: $u_{\text{e}} = u_0 \cos\alpha$
d_{e}	Effective diameter of the droplet initiating the off-center collision where the interaction region is seen as a spherical droplet: $d_{\text{e}} = d_0 (1-B)^{\frac{2}{3}} (1+2B)^{\frac{1}{3}}$
r_{e}	Effective radius of the droplet initiating off-center collision: $r_{\text{e}} = \frac{d_{\text{e}}}{2}$
h	Overlapping interaction region between colliding droplets: $h = 2r_0(1-B)$
π	Circumference ratio

g	Acceleration of gravity
β	Slope angle of the tilted substrate
ω	Minimum tilt angle required for the droplet to roll-off a substrate on a slope: $\omega = \arcsin \frac{3f}{4\pi r_0^3 \rho g}$
f	Friction between the droplet and the contacting solid surface: $f = \frac{4}{3}\pi r_0^3 \rho g \sin\omega$
a	Acceleration of a droplet that rolls off an ideal slope (friction-free): $a = g\sin\beta$
a'	Acceleration of a droplet that rolls off a practical slope: $a' = \frac{2s}{t^2}$
s	Distance that the droplet rolls on the slope over time t
t	Time of the droplet rolling on the slope over distance s
P_c	Capillary pressure of a droplet impacting on a porous surface: $P_\mathrm{c} = -\frac{2\gamma\cos\theta_\mathrm{a}}{d_\mathrm{pore}}$
θ_a	Advancing liquid contact angle of a flat surface
d_pore	Average pore diameter of a porous surface
P_d	Dynamic pressure of an impacting droplet: $P_\mathrm{d} = \frac{\rho u_0^2}{2}$
P_h	Effective liquid hammer pressure: $P_\mathrm{h} = \frac{\rho c u_0}{5}$
c	Velocity of sound in a liquid
e	Coalescence efficiency of droplet–droplet collisions
τ_e	Oscillation period when considering the interaction region where the colliding droplets (d_0) overlap as a spherical droplet with a diameter of d_e: $\tau_\mathrm{e} = \sqrt{\frac{\rho d_\mathrm{e}^3}{\gamma}}$
τ_d	Portion of an oscillation period where the bouncing droplets are compressing: $\tau_\mathrm{d} = \tau_0 - \tau_\mathrm{r}$
τ_r	Portion of an oscillation period where the bouncing droplets are separating: $\tau_\mathrm{r} = \tau_0 - \tau_\mathrm{d}$

$L(t)$	Droplet deformation as a function of time
i	Number of droplets in the droplet collision system
DMF	*N*,*N*-Dimethylformamide
SDS	Sodium dodecyl sulfate
PEO	Polyethylene glycol
PMMA	Poly(methyl methacrylate)
NaCl	Sodium chloride
H_2O	Water

Methods

Super-repellent surface. A piece of copper mesh (McMaster-Carr; 100 × 100, i.e. number of openings per 1 × 1 in^2) was first spray-coated with 10 mg mL^{-1} solutions of *n*-butyl cyanoacrylate (BOC Sciences) + 20 wt.% 1*H*,1*H*,2*H*,2*H*-perfluorohexyltrichlorosilane (Gelest) in Asahiklin 225 (Asahi Glass Co.) for ca. 5 min using a Paasche airbrush at a distance of ~20 cm with a N_2 pressure of 58 psi and subsequently cross-linked in an oven at 70 °C for 2 h. The substrate was stored in a dust-free desiccator, and any unbound particles on the super-repellent coating were removed using compressed air before the coated mesh super-repellent surface was subjected to the droplet collision experiments.

Characterization. A Philips XL30 scanning electron microscope was used to image the surface morphology of the super-repellent surface at 5 kV. A Ramé-Hart 200-F1 goniometer was used to measure contact angles, roll-off angles and liquid surface tension. Averages from $N \geq 5$ independent measurements were reported and the measured errors in contact angle, roll-off angle and surface tension were ±1°, ±0.5° and ±1 mN m^{-1}, respectively. The surface durability was demonstrated by continuous droplet impinging and rolling tests using 1 mM SDS aqueous droplets. Liquid droplets were released from a height of 10 cm above the substrate and rolled off after impinging on the surface. The roll-off angle was reassessed after a set numbers of tests (Figure S1). High-speed movies were obtained using a Fastec Hispec1 camera.

Droplet rolling. We determined the friction between the super-repellent copper mesh and the contacting liquid by droplet roll-off on a slope. The minimum tilt angle ω required for droplet roll-off was experimentally demonstrated to be <3° for all liquids tested (Table S1), thus the friction was determined as 10^{-7}–10^{-6} N by balancing with the gravity force which gives $f = \frac{4}{3}\pi r_0^3 \rho g sin\ \omega$ (see details in *Abbreviations and Definitions*). Such minute forces enable the droplets to roll on the surface in a way close to the ideal case, which could be concluded by

comparing the accelerations as a mass body (i.e., liquid droplet) rolling off a given slope $\beta > \omega$. For the ideal case, the acceleration is given as $a = g\sin\beta$. By recording the distance s that a droplet travels in a period t on a super-repellent slope β, the practical acceleration a' along the slope can be computed as $a' = \frac{2s}{t^2}$. When a liquid droplet (e.g., n-pentane) rolls off a surface tilted at 37°, the measured acceleration was 5.85 m s^{-2}, in good agreement with the ideal case (5.90 m s^{-2}). The near-zero friction super-repellent surface used herein thus provides a suitable platform for studying collisions between droplets moving in two dimensions on the platform, as well as the droplet–droplet bouncing events.

Droplet bouncing. When a droplet impacts on a horizontally placed super-repellent surface, the droplet experiences dynamic deformations and bounces off the substrate after transient contact. The robustness of the surface superrepellency upon droplet impact can be investigated by balancing the capillary pressure generated within the surface textures, the dynamic pressure created by the initial impinging droplet and the effective liquid hammer pressure occurring at the spreading stage.[S1] The capillary pressure is defined as $P_c = -2\gamma\cos\theta_a/d_{pore}$, where θ_a and d_{pore} are local advancing contact angle (which equals to that of a flat surface) and the average pore diameter of the coating, respectively; the dynamic pressure is defined as $P_d = 0.5\rho u_0^2$; and the effective liquid hammer pressure is defined as $P_h = 0.2\rho c u_0$, where c is the sound velocity in the liquid. When a water droplet impinges on the super-repellent surface from a height of 10 cm, P_c, P_d and P_h are estimated as 0.7, 0.001 and 0.1 MPa, respectively. These values indicate that the capillary pressure of the nano-scale coating outweighs the wetting pressures. The robustness of the coating thus allows broad impacting conditions useful for droplet collision experiments.

Droplet collision. A super-repellent copper mesh (10 cm × 5 cm) was bent at the midway point to have a slope of ~37° for droplet acceleration followed by a flat section for collisions (Figure S2). Shallow depressions and tracks (~0.1 mm) were forged by pressing with a metal ball to

enable the deposition of droplets and to guide the direction of the rolling droplets. A droplet was first placed in a depression and another droplet was subsequently released on the slope. Droplet collisions were recorded vertically from above the surface using a manually triggered camera at 5,155 frames per second (unless specified otherwise). Photron FASTCAM viewer software was used to analyze the droplet size r_0, impact parameter B and impact velocity u_0 upon collisions, and the droplet deformation and contact time for bouncing collisions.

Collision boundary. Three collision regimes can be defined as (1) bouncing collision, (2) permanent coalescence and (3) stretch separation (Figure S3 and S4). At high impact parameters, only a small region of the droplets will come into contact: the width of the interaction region can be computed as $h = 2r_0(1 - B)$ if the colliding droplets are identical. Thus, the boundary between permanent coalescence and stretch separation can be computed by balancing the total effective stretching kinetic energy and the surface energy of the interaction region:[S2]

$$We = \frac{16\left[3(1-B)(1-B)^2(1+2B)\right]^{\frac{1}{2}}}{1-(1-B^2)(1-B)^2(1+2B)} \tag{S1}$$

As is shown in Figure S5, though Equation S1 is applicable for droplet collisions in air, the prediction works reasonably well with other experimental results even for our collision study on a solid surface. This indicates that the porous super-repellent platform makes the droplet collision acts as if it took place in air. In contrast, when droplet collision occurs on a non-porous surface,[S3] film drainage slows as the air flow is hindered, which results in a high probability of bouncing collisions.

By comparison, the occurrence of bouncing collisions is largely dependent on the maintenance of the layer of air, which originates from the local dynamics of the fluid (e.g., air and droplet). A possible criterion for bouncing is that the effective kinetic energy only produces limited deformation of the colliding droplets:[S4]

$$We = \frac{2.8}{\left(1-B\right)^2 \left(1+2B\right)\left[\cos\left(\arcsin B\right)\right]^2} \qquad \text{(S2)}$$

Though our observations are mostly within the range predicted by this criterion, understanding the mechanism of maintaining a vapour layer remains challenging.[S5] In some cases, the probabilities of bouncing and coalescence are comparable. For perspective, a higher chance of bouncing collisions requires a more repulsive interaction interface, and a locally high air pressure may prevent permanent coalescence.[S6]

As for collisions of binary composition droplets (i.e., identical size), same regimes can be observed (Figure 1g, Figure S4). The boundaries of bouncing, coalescing, and stretch separation can also be described by the above Equation S1 and Equation S2, respectively. The *We* of these events can also be determined by the moving droplets on the super-repellent platform.

In cases including pesticide spraying, which is usually through an aqueous media, and spray coating, which requires either an organic or an aqueous solvent, coalescence in the sprayed droplets is desirable. We thus investigated the coalescence efficiency of droplet collisions on the super-repellent surface (Figure S6). The coalescence efficiency *e* of polar aqueous droplets was experimentally determined to be directly related to the impact Weber number (i.e. $e \sim \frac{3}{We}$). This result is in good agreement with droplet collisions in air.[S7] However, non-polar droplets, such as *n*-pentane, behave completely differently within the same Weber number range. This might be due to the comparably weaker intermolecular forces of non-polar liquids, which result in a higher chance to bounce off before coalescence.

Maximum deformation. At the time corresponding to roughly half of the oscillation period (i.e., ~$0.5\tau_0$), the bouncing droplets reach maximum deformation l_{max}, which is due to the excess of inertial forces over the surface tension of the droplet, similar to the case observed for droplets impacting on a super-repellent surface,[S8,S9] where maximum deformation occurs at ~$0.3\tau_0$ due

to the non-compensatory blocking exerted by the non-movable hard-solid super-repellent surface. In comparison, the droplet–droplet interfaces during bouncing collisions are much more flexible considering the soft nature of liquid surfaces, as well as the high mobility of droplets on the near-zero friction super-repellent platform. However, gravity plays a negligible role in horizontal collisions (e.g., droplet–droplet bouncing collisions). In contrast, for a bouncing droplet impacting vertically on a solid surface (i.e., single droplet bouncing collision), the vertical impact force is in the same direction as the gravitational force, which allows the droplet to deform to a greater extent (~10%) (Figure 2b).

Effective parameters. For off-center collisions, droplet–droplet interaction mainly occurs within a reduced region, while the other regions of each droplet tend to maintain the initial trajectory of the droplet. Therefore, there is a practical need to define effective parameters to quantify the bouncing dynamics of off-center collisions. As is illustrated in Figure 3b, off-center collisions can generally be categorized into three types of head-on collisions. Case I considers the droplet head-on collision using effective velocity (u_e = $u_0$cos α. Case II considers two smaller droplets with effective dimensions ($r_{\mathrm{e}} = \frac{d_{\mathrm{e}}}{2} = r_0(1-B)^{\frac{2}{3}}(1+2B)^{\frac{1}{3}}$) colliding at a speed of u_0 (i.e., the volume of the droplet is equivalent to that of the interaction region). Case III considers both effective parameters (velocity and volume) at the same time. As a consequence, all three cases should be considered in the calculation of Weber number (i.e. $We_1 = \frac{\rho r_0 u_{\mathrm{e}}^2}{\gamma}$, $We_2 = \frac{\rho r_{\mathrm{e}} u_0^2}{\gamma}$, $We_3 = \frac{\rho r_{\mathrm{e}} u_e^2}{\gamma}$). However, the effective oscillation period of the interaction region can be simply written as $\tau_{\mathrm{e}} = \sqrt{\frac{\rho d_{\mathrm{e}}^3}{\gamma}}$.

Contact time. First, we consider droplet–droplet head-on bouncing collisions analytically. Bouncing droplets' transient contact is composed of two parts—deformation (τ_d) and retraction (τ_r)—that are expected to be functions of the droplet oscillation period (i.e., $\tau_0 = \tau_{\mathrm{d}} + \tau_{\mathrm{r}}$).

According to a study on binary liquid collisions,[S10] the timescale for maximum droplet deformation can be empirically determined to be $\tau_d \approx 0.5\tau_0$ for low Weber numbers (i.e. *We* < 20). Based on the oscillation profile of the droplet, the retraction time scales to approximately half of the oscillation period as well ($\tau_r \approx 0.5\tau_0$). Thus, for head-on bouncing collisions, the contact time follows the oscillation law. For small *We* (*We* << 1), the contact time nearly doubles (Figure 2d) as the kinetic force is too weak to overcome the surface energy of the droplet, and the mobility of the droplet becomes more restricted as the friction work done by the super-repellent surface becomes non-negligible relative to the small kinetic energy. Three scaling methods can be considered: the classical inertia-capillary scaling (i.e., by neglecting the fluid viscosity), the viscous scaling (i.e., fluid's viscosity is not negligible), and the visco-capillary scaling (Figure S7). For the present work, the viscosities of the probing liquids are mostly <10 mPa s, which are considerably small. And the inertia-capillary timescale enters the scaling of the contact times of bouncing droplet–droplet head-on collisions. Considering the impact velocities of the moving droplets mostly less than 1 m s^{-1} in the present work, the contact time of the horizontal bouncing droplet–droplet collision is also independent on the impact velocity (Figure S8 and S9), which is similar to the case of the vertical droplet bouncing on super-repellent surfaces (Figure S10), as well as the vertical droplet bouncing on another droplet residing on the super-repellent surface (Figure S12).

For off-center bouncing collisions, the contact time scales as the volume of the interaction region (Figure 3d, Figure S11). Taking into account the effective droplet diameter, we can deduce the contact time for off-center bouncing collisions as:

$$t_c \approx \tau_e = \sqrt{\frac{\rho d_0^3 (1-B)^2 (1+2B)}{\gamma}} = \tau_0 (1-B)(1+2B)^{1/2} \quad \text{(S3)}$$

Oscillator. Two droplets involving bouncing collisions can be seen as oscillators, in which their deformation can be written as $L_1(t)$ and $L_2(t)$, respectively. For identical droplet collisions, both droplets undergo similar deformation (i.e. $L_1(t) = L_2(t)$) and the contact time can be estimated by the droplet oscillation period as $t_c \approx \tau_1 = \tau_2$. As for the contact time of binary liquid droplet–droplet bouncing, theoretically we have to consider the expansion rate and the retraction rate in the precinct of droplet departure. For example, considering the head-on bouncing collision of a water–hexadecane system (i.e., a water droplet impacts onto a hexadecane drop), at the end of contact, the water droplet experiences retraction while the hexadecane droplet is still expanding. After the time point where these two events reach the same rate, the two droplets depart from each other permanently. Thus, the contact time can be computed as $\frac{\partial L_1(t)}{\partial t} + \frac{\partial L_2(t)}{\partial t} = 0$. The timescale determined this way falls within the range of $\tau_1 \sim \tau_2$ and is actually closer to the longer oscillation period (Figure 2f).

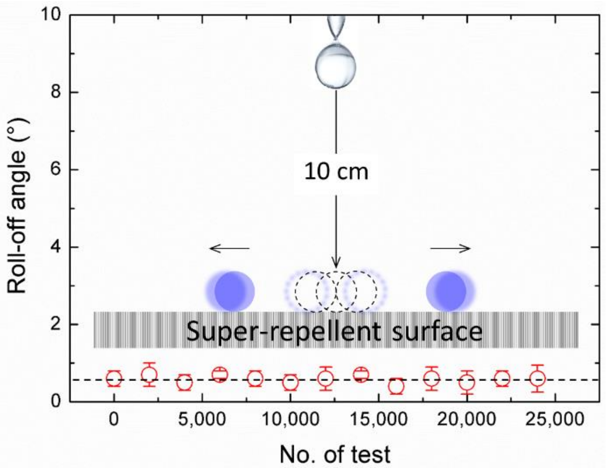

Figure S1. Durability of the super-repellent copper mesh coated by spraying a mixture of fluoro-silanes and cyanoacrylate. *Super-repellent surface*: Dimensions of the woven fiber and the inter-fiber spacing after coating are 123 and 143 μm, respectively. Average pore size of the super-repellent coating is within the nanoscale range (100–1,000 nm). *Impact durability test*: The impacting droplets (1 mM SDS) were generated using a syringe pump and released at a height of ~10 cm above the substrate. Roll-off angles of droplets remained constant even after 20,000 cycles of droplet impinging, bouncing, and rolling tests. The error bars represent standard deviations obtained from $N \geq 5$ independent measurements.

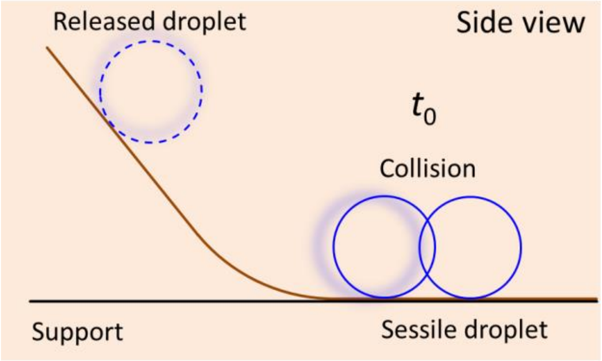

Figure S2. Schematic of droplet–droplet collision occurring on a super-repellent solid surface. Liquid droplet released on the super-repellent slope impacts on the stationary droplet, which leads to bouncing, coalescing, or stretch separation. The impact velocity of the moving droplet can be tuned by adjusting the releasing position on the friction-free super-repellent surface. The incidental collision angle can be tuned by adjusting the placement of the stationary droplet along the track of the moving droplet.

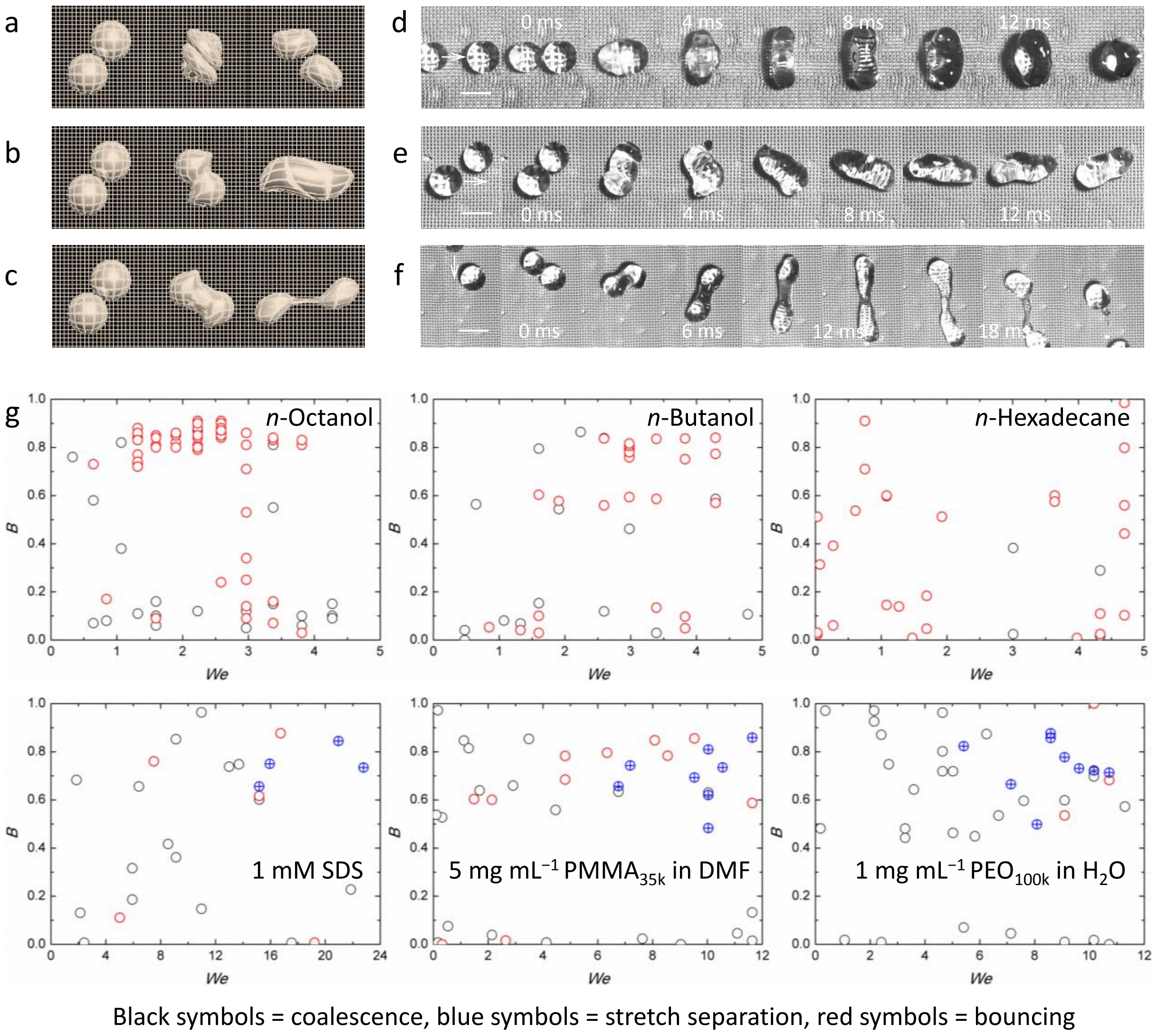

Figure S3. Collision regimes of single liquid droplet–droplet systems. a–c, Schematics of bouncing (a), coalescence (b) and stretch separation (c) of single composition droplet–droplet collisions, respectively. d–f, Snapshots of coalescing and stretch-separating collisions of 1 mM SDS aqueous droplets shown in Video S4–S6: head-on permanent coalescence (d); off-center permanent coalescence (e); off-center coalescence followed by stretch separation (f). Scale bars are 2 mm. g, Collision regimes of single liquid droplet–droplet systems within collision angles of 0–90° and analytical boundaries showing bouncing (red), coalescence (black) and stretch separation (blue) events. $B = \sin\alpha$ is impact parameter. $We = \frac{\rho r_0 u_0^2}{\gamma}$ is Weber number, where r_0, ρ, γ and u_0 are the initial radius, density, surface tension and impacting velocity of the droplet, respectively. The droplets are *n*-octanol, *n*-butanol, *n*-hexadecane, 1 mM SDS, 5 mg mL^{-1} PMMA$_{35k}$ in DMF, and 1 mg mL^{-1} PEO$_{5000k}$ in H_2O. See Table S1 for more details of liquids used.

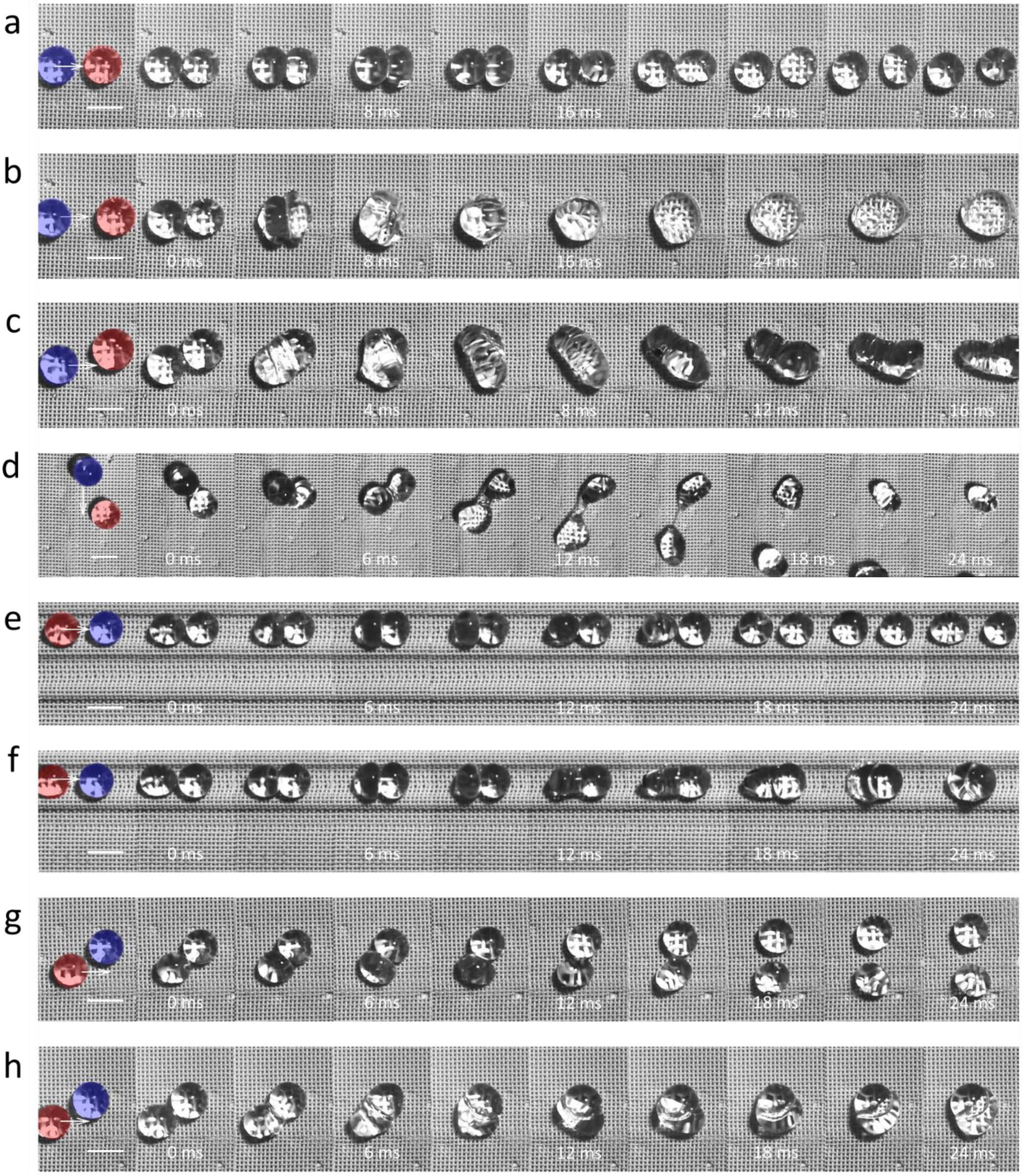

Figure S4. Outcomes of binary liquid droplet–droplet collisions. Collision regimes when a water droplet impacts onto a hexadecane droplet: a, head-on bouncing; b, head-on coalescence; c, off-center coalescence; d, off-center stretch separation. Collision regimes when a hexadecane droplet impacts onto a water droplet: e, head-on bouncing; f, head-on coalescence; g, off-center bouncing; h, off-center coalescence. Scale bars are 2 mm.

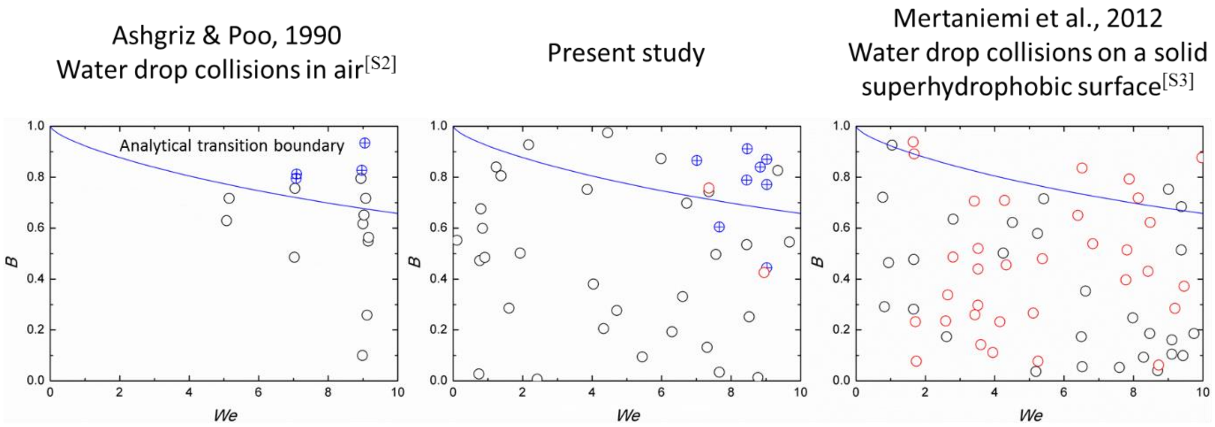

Black symbols = coalescence, blue symbols = stretch separation, red symbols = bouncing

Figure S5. Water collision regimes with theoretical predictions. Droplet bouncing collision, coalescing collision and stretch separation are represented by red, black and blue symbols, respectively. The blue line is the analytical transition boundary.[S2] The present study using a porous super-repellent surface resembles the case of droplet collisions in air but differs from the work performed on a non-porous superhydrophobic surface.[S3] The porosity of the super-repellent coating enables the collisions to behave similarly to in-air collisions[S2] by providing free air flow at the contacting interface of the droplets and a thin air cushion underneath the droplet. These events also demonstrate the extreme repellence of the substrate to liquids. It is also noted that the droplet–droplet collisions might be unique or an extreme scenario to the case where a droplet impacts on a liquid surface.[S11]

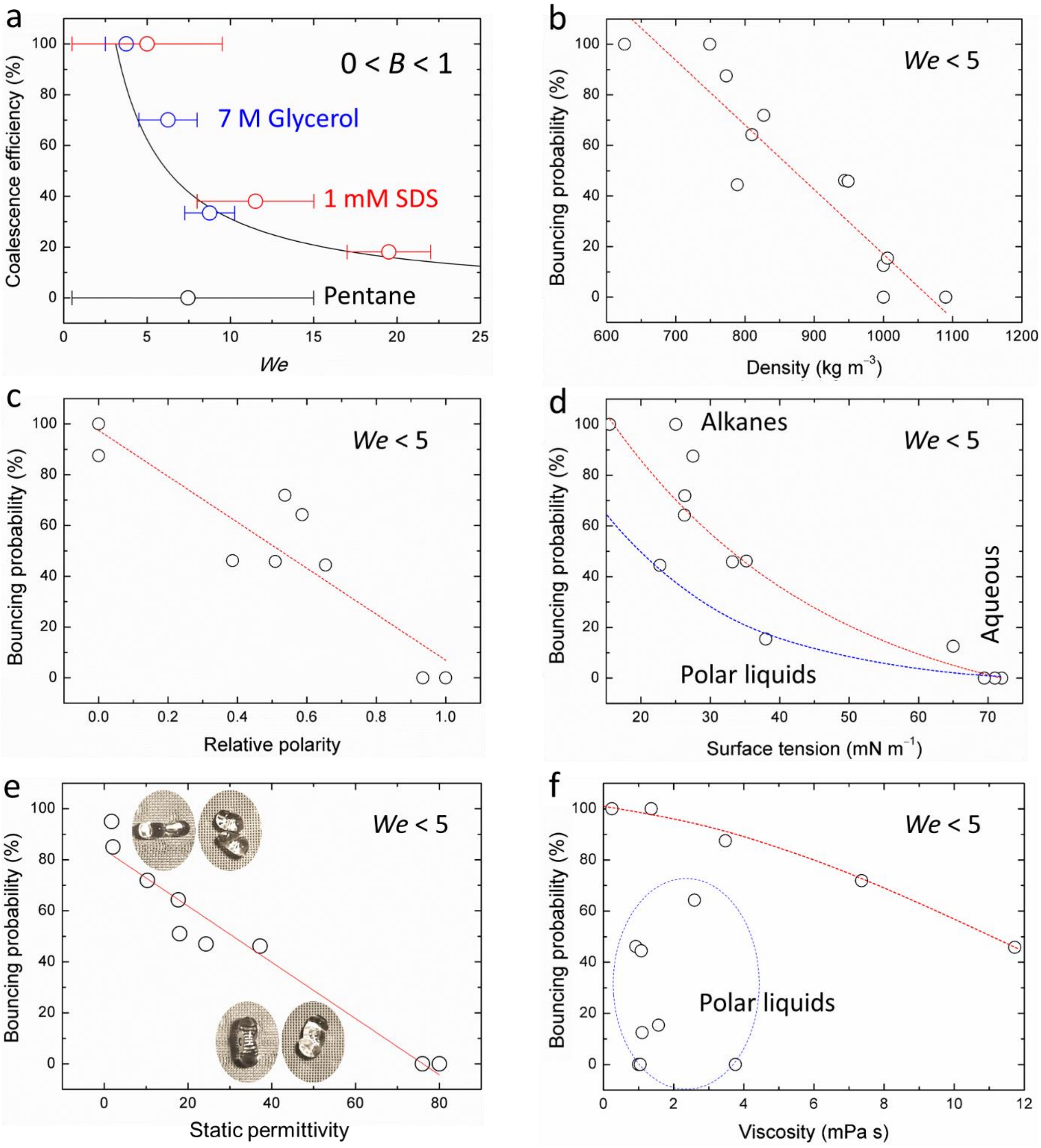

Figure S6. Coalescence efficiency and bouncing probability. a, Coalescence efficiency of polar aqueous solutions and non-polar alkane droplets. The symbols represent the average coalescence efficiency of at least 10 independent measurements in the corresponding *We* range indicated by the error bars. Probability of bouncing collisions against liquid density (b), relative polarity (c), surface tension (d), liquid static permittivity (e) and liquid viscosity (f). The order of liquid permittivity in (e) is *n*-pentane < *n*-hexadecane < *n*-octanol < *n*-butanol < cyclopentanol < ethanol < dimethylformamide < 0.5 mM aqueous SDS < water. Insets in (e) are

representative outcomes for low- (top: head-on and off-center bouncing) and high-permittivity (bottom: head-on and off-center coalescence) colliding liquids. Liquids that have a low static permittivity have a higher probability to bounce, whereas liquids that have a high static permittivity have a higher probability to coalesce. This is likely related to the dispersion forces within the liquids.[S12]

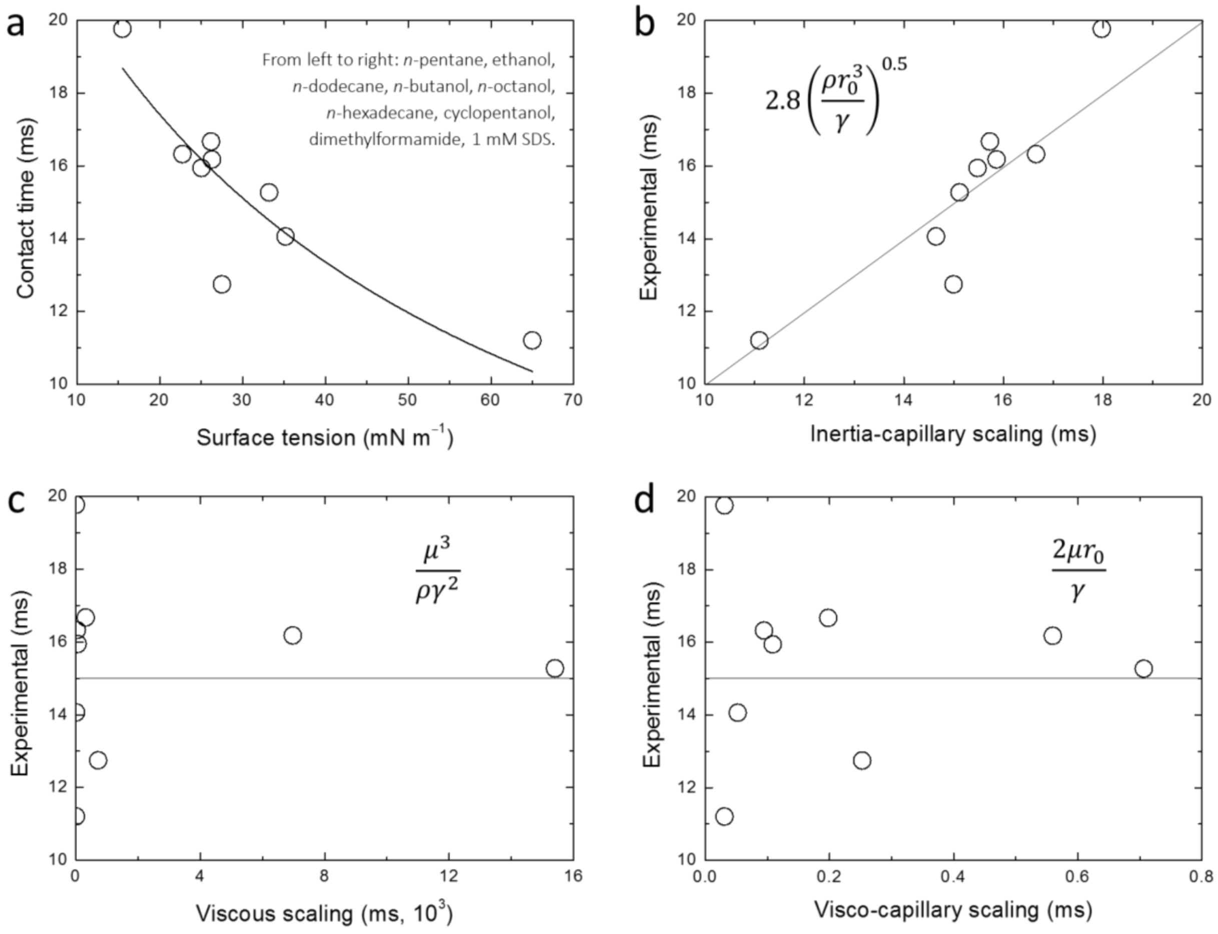

Figure S7. Scaling the contact time of droplet–droplet bouncing. a, Experimental contact times plotted against the liquid surface tension. b, Inertia-capillary scaling method. c, Viscous scaling method. d, Visco-capillary scaling method. The inertia-capillary timescale matches well with the experimental results for the present work, considering the probing liquids tested are mostly non-viscous (i.e., μ <10 mPa s).

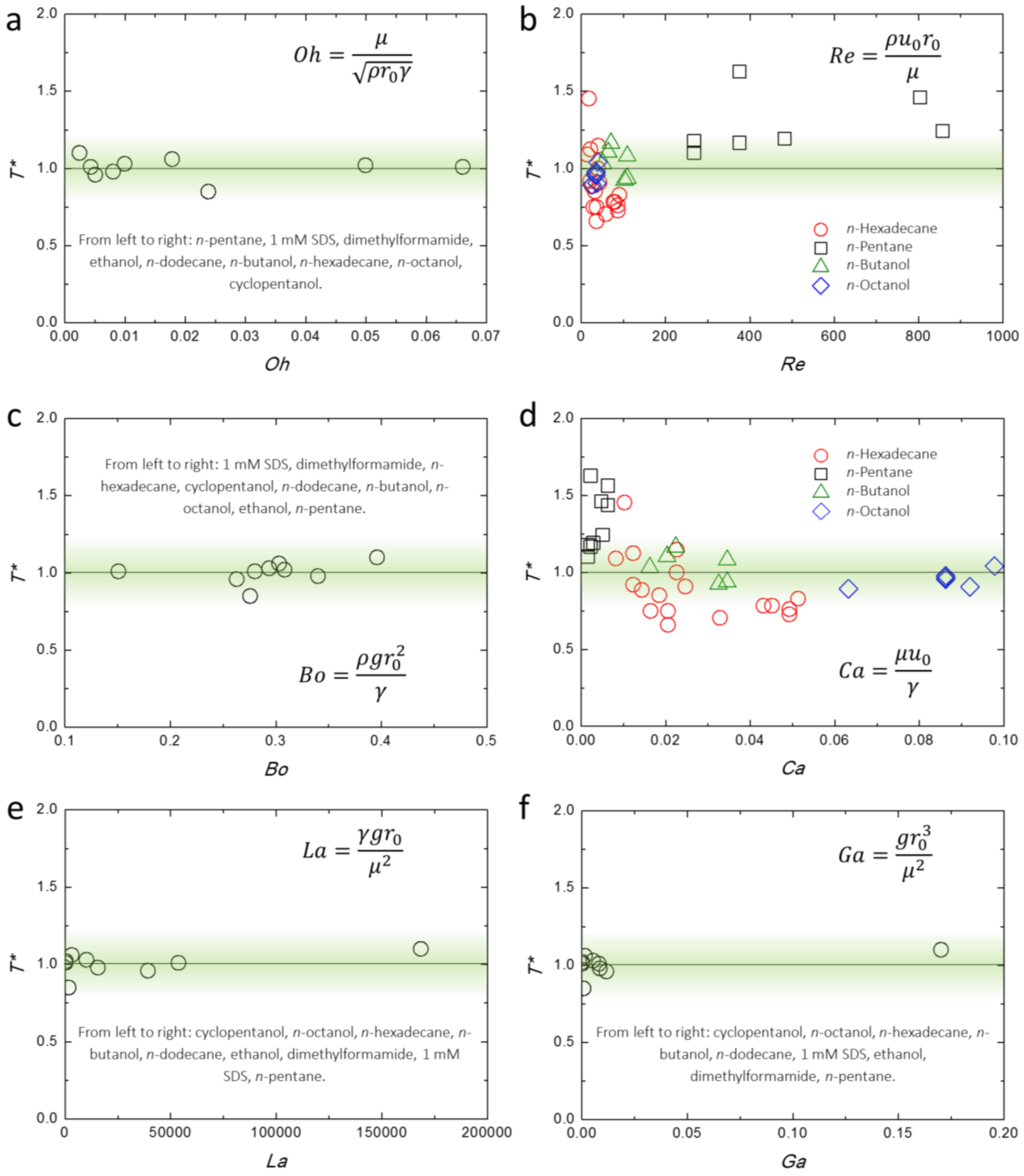

Figure S8. Contact times of head-on droplet–droplet bouncing collisions against various dimensionless parameters. The contact times are normalized by the corresponding inertia-capillary timescales of the liquids. Dimensionless parameters considered here include a, Ohnesorge number *Oh*, b, Reynolds number *Re*, c, Bond number *Bo*, d, Capillary number *Ca*, e, Laplace number *La*, f, Galilei number *Ga*. See details in the section *Abbreviations and Definitions*. The independence of the contact times to these dimensionless parameters indicates that the inertia-capillary scaling methods hold within these fluidic conditions outlined.

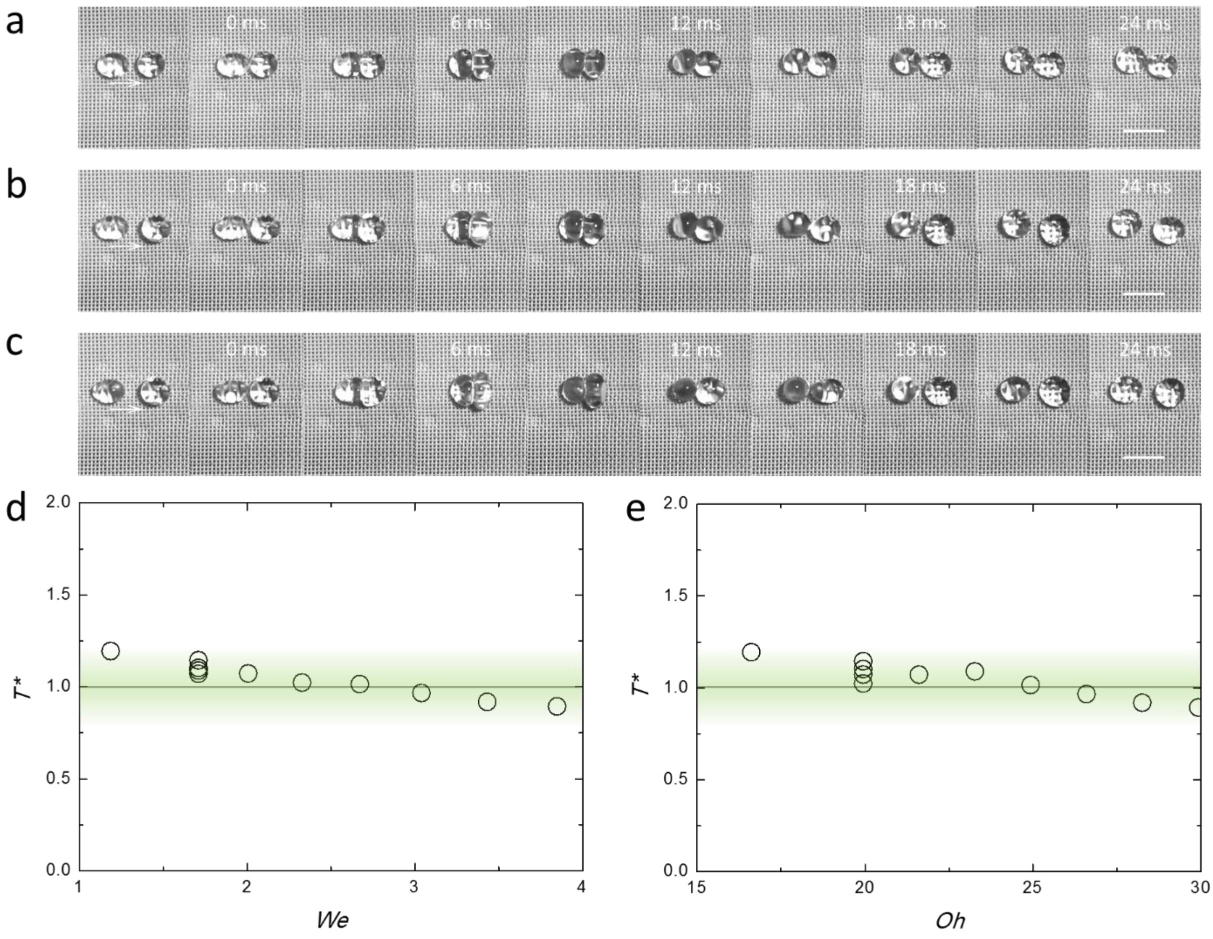

Figure S9. Head-on bouncing collisions of cyclopentanol droplets. The impact Weber numbers in a–c are 1.2, 2.3 and 3.4, respectively. Droplet deformation increases as *We* increases, whereas the contact time remains constant and scales as its oscillation period. Scale bars are 2.5 mm. The dimensionless contact times are also plotted against the impact Weber number (d) and the Ohnesorge number (e). As is shown, slightly decreased contact times are observed but they are still largely scaled with the corresponding inertia-capillary timescales. This might be due to the slightly higher viscosity of cyclopentanol droplets (11.7 mPa s) and the viscous dissipation might start to play a part in determining the contact time of bouncing droplet–droplet collisions.

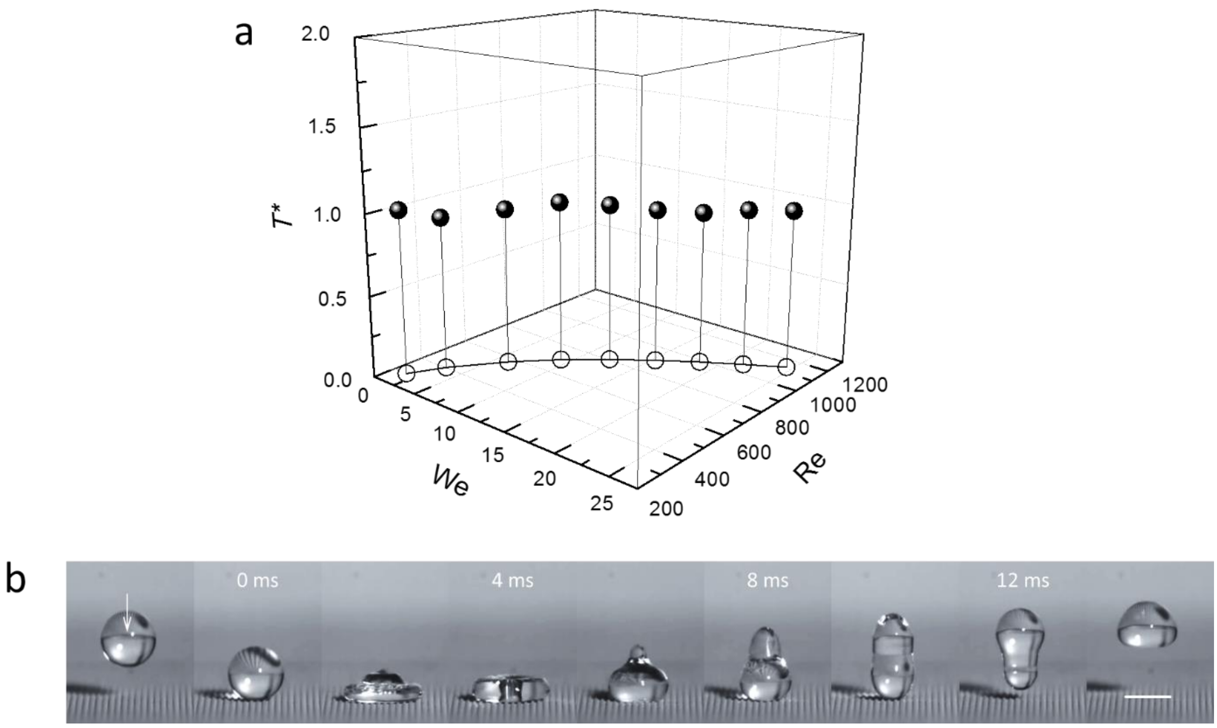

Figure S10. Contact time of droplet–solid surface bouncing collisions. The droplets are of 1 mM aqueous SDS. a, The contact time shows independence to the dimensionless parameters (i.e., *We*, *Re*). b, Snapshots of a bouncing droplet of 1 mM SDS on the super-repellent copper mesh. The contact time t_c is 10–11 ms, in consistent to the inertia-capillary timescale τ_0. Scale bar is 2 mm.

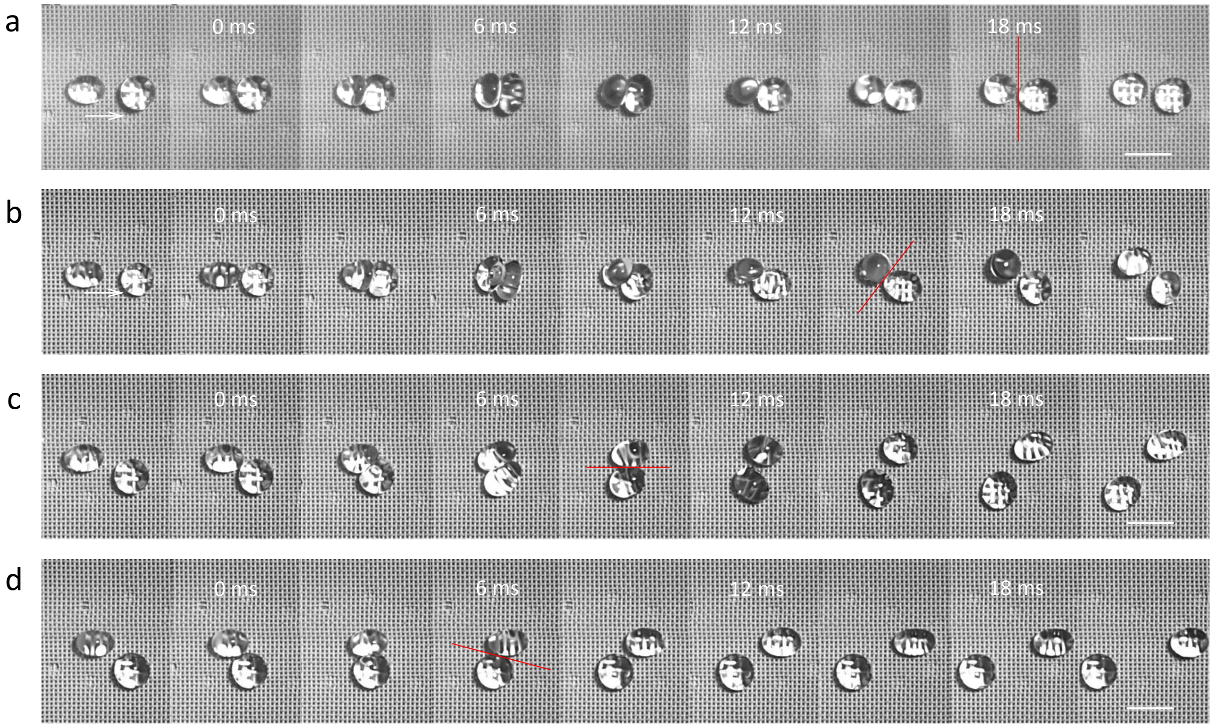

Figure S11. Dynamics of bouncing collisions with varied impact parameters. Through a–d, the impact parameter of colliding *n*-octanol droplets increases from 0.0 to 0.1, 0.5 and 0.7, respectively. Scale bars are 2.5 mm. The red lines represent the point of separation between two droplets. The contact time decreases as the impact parameter increases.

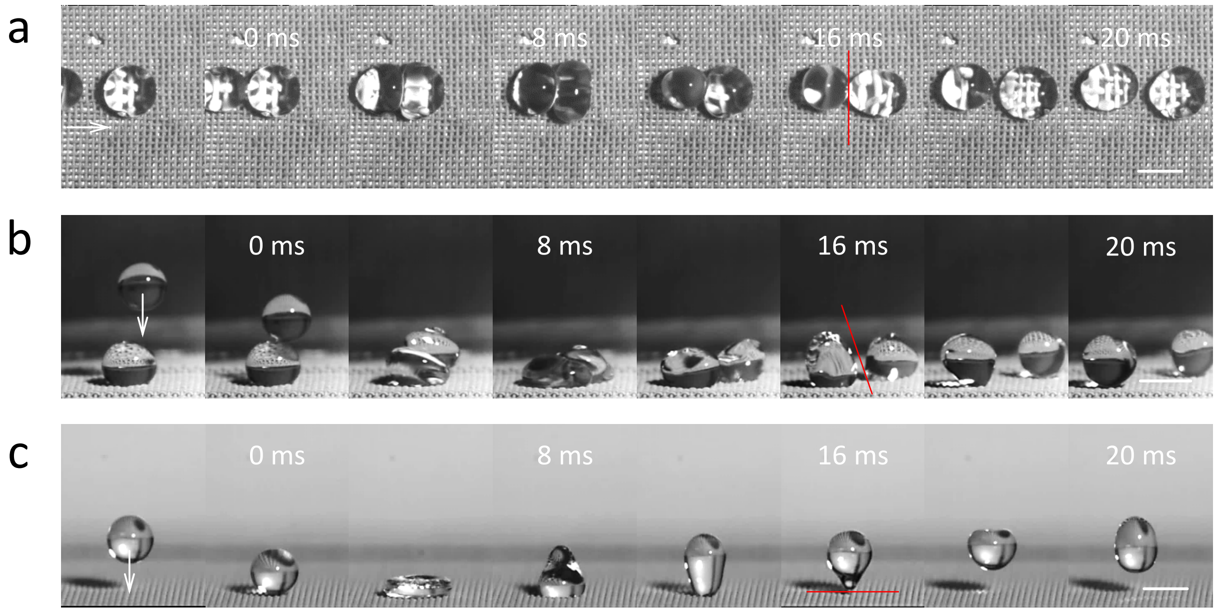

Figure S12. Unification of droplet bouncing demonstrated by hexadecane droplets. a, Head-on droplet–droplet collision on a super-repellent surface. b, Near head-on droplet collision with the moving droplet impacting vertically onto the other droplet supported by a super-repellent surface. c, A hexadecane droplet bouncing on a super-repellent surface. Scale bars are 2 mm. The three droplet bouncing scenarios result in the same contact time ($t_c \approx 16$ ms). The red lines indicate the point of separation between two droplets or a droplet and the surface. See Table S2 and S3 for more information.

Table S1. Surface super-repellency of liquids examined in the study

Liquid	Surface tension (mN m^{-1})	Advancing contact angle (°)	Receding contact angle (°)	Roll-off angle (°)
Water	72.0	172	171	0.5
1 M NaCl in water	72.3	172	170	0.5
7 M Glycerol in water	69.5	172	170	0.5
1 mg mL^{-1} PEO_{100k} in water	64.0	171	168	1.0
0.5 mM SDS in water	71.0	172	170	0.5
1 mM SDS in water	65.0	170	169	0.5
200 mM SDS in water	38.0	168	165	1.0
DMF	35.2	166	162	2.0
5 mg mL^{-1} $PMMA_{35k}$ in DMF	35.8	167	162	2.5
Cyclopentanol	33.2	165	162	2.0
n-Octanol	26.3	165	161	2.5
n-Butanol	26.2	163	160	2.0
Ethanol	22.7	163	159	3.0
n-Hexadecane	27.5	162	159	2.0
n-Dodecane	25.0	161	158	3.0
n-Pentane	15.5	160	156	3.0

Surface tension values (at 25°C) were obtained from the pendant drop method mentioned in *Characterisation* in *Methods*. DMF, dimethylformamide; SDS, sodium dodecyl sulfate; PMMA, poly(methyl methacrylate); PEO, polyethylene oxide. Subscripts (i.e. M in $PMMA_M$) indicate the average molecular weight M_w.

Table S2. Droplet bouncing mediated by a vapour layer

Droplet bouncing system	Platform type	Droplet	T^{*a}	Reference
Droplet oscillation (2 phases)	Air	Arbitrary	1.00	S13
Droplet–solid collision (3 phases)	Hot solid	Water	0.85	S14
	Wetting solid	Arbitrary[b]	0.92–1.07	S5
	Hydrophobic solid	Water	0.72	S15
	Superhydrophobic solid	Water	0.95	S16,S17
	Superoleophobic solid	Hexadecane	1.04	S18
	Superomniphobic solid	PDMS	0.80	S19
	Reentrant texture	FC-72	1.38	S20
	Tapered posts	Water	0.90	S21
	Curved solid	Water	0.92	S22
	Macrotexture ridge	Water	0.74	S23,S24
Droplet–liquid collision (3 phases)	Oscillated liquid bath	Silicon oil	1.90	S25
	Thin liquid film	Water	0.97	S26
	Liquid bath	Water	1.20	S27
Droplet–droplet collision (3 phases)	Air	Hydrocarbon	0.94	S6
	Sessile droplet	Water	1.25	S28
Droplet–droplet collision (4 phases)	Free droplet on solid	Water	1.26	S29
	Superhydrophobic solid	Water	1.00	S3
**Droplet–droplet collision (4 phases)**	**Super-repellent solid**	**Arbitrary (16 liquids)[c]**	**0.84–1.16**	**Present work**
		Water	1.04	
		1 M NaCl	0.98	
		7 M Glycerol	0.92	
		$PEO_{100\,k}$[d]	1.05	
		0.5 mM SDS	1.02	
		1 mM SDS	1.01	
		200 mM SDS	0.97	
		DMF	0.96	
		$PMMA_{35\,k}$[e]	1.09	
		Cyclopentanol	1.01	
		n-Octanol	1.02	
		n-Butanol	1.06	
		Ethanol	0.98	
		n-Hexadecane	0.84	
		n-Dodecane	1.03	
		n-Pentane	1.16	
Droplet–droplet collision (>5 phases)	Super-repellent solid	Arbitrary	–	Future work

[a]The contact time T^* is non-dimensionalised against the effective oscillation period $(\rho d_e^3/\gamma)^{0.5}$. [b]The liquids include water, 85 wt.% glycerol, sunflower oil, 90 wt.% propanol, decane, silicone oil, and FC-40. [c]See Table S1 for specific details. [d]1 mg mL^{-1} PEO_{100k} in water. [e]5 mg mL^{-1} $PMMA_{35k}$ in DMF. PDMS, polydimethylsiloxane; FC-72, perfluorohexane.

Table S3. Summary of droplet bouncing studies

Entry	Bouncing droplets	Impinged materials	*T**	Reference
1	Water	Hydrophobic/superhydrophobic silicon surfaces with square arrays of pillars	0.90–0.99	S30
2	Water	Superhydrophobic surfaces with different morphologies and roughness	0.62–1.13	S31
3	Water	Filamentary superhydrophobic Teflon surfaces	0.84–1.18	S32
4	Water	Hierarchical structured superhydrophobic surface	0.94–1.14	S33
5	Water	Painting with perfluorosilane-coated titanium dioxide nanoparticles	0.91–1.04	S16
6	Water	Heated hydrophobic and hydrophilic surfaces	1.04–1.42	S34
7	Water	Hydrophobic surfaces with microcavities	0.72–1.14	S35
8	Water	Stretchable superhydrophobic surfaces with hierarchical wrinkles	1.05–1.59	S36
9	Water<br>Ethylene glycol<br>Cyclohexanone<br>Xylene<br>Toluene	Transparent and flexible superoleophobic nanotextures	0.98–1.12	S37
10	Water	*B. oleracea* L. leaf and other superhydrophobic surfaces	0.67–1.27	S38
11	Water	Lotus leaf and superhydrophobic diarylethene microcrystalline surfaces	0.98–1.06	S39
12	Water	Perfectly hydrophilic surfaces	0.72–0.85	S40
13	Water	Rigid superhydrophobic surface in a low-pressure environment	0.83–1.25	S41
14	Water	A thin layer of perfluorinated fluid Dupont Krytox oil	0.85–0.93	S26
15	Water	Superhydrophobic macrotextures	0.78–1.02	S23
16	Water	Curved superhydrophobic surfaces	0.56–0.88	S22
17	Water	Superhydrophobic surfaces patterned with a square lattice of tapered posts	0.17–0.90	S21
18	Water<br>85% Glycerol<br>Sunflower oil<br>90% Propanol<br>Decane<br>Silicone oil<br>FC-40	Wetting and non-wetting flat surfaces	0.70–1.58	S5
19	Water	Rigid and flexible superhydrophobic surfaces	0.42–1.60	S42
20	Water	Elastic superhydrophobic surfaces	0.75–0.94	S43

21	Water	Superhydrophobic porous networks	0.25–0.93	S44
22	Water	Superhydrophobic solid surfaces	0.90–0.93	S17
23	Water	Nanostructured superhydrophobic surfaces cooled down to –15°C	0.93–1.24	S45
24	Water	Superhydrophobic surface	0.83–0.92	S9
25	Water	Superhydrophobic substrate	0.94	S46
26	Water	Micropatterned superhydrophobic surfaces	1.42	S47
27	Water	Hydrophobic surfaces	1.58	S48
28	Water	Superhydrophobic solid surface	0.92–1.00	S49
29	Water	A hot plate over 400 °C	0.51–0.96	S50
30	Molten tin	A stainless–steel surface at 240 °C	0.87–1.11	S51
31	Toluene	Water–solid interface	0.75–1.73	S52
32	Water	Microtextured superhydrophobic materials	0.94–1.08	S53
33	Water	Micropatterned superhydrophobic silicon surface	1.52–1.90	S54
34	Water/glycerin mixture	Superhydrophobic silicon nanowires	0.93	S55
35	Hexadecane	Silanized microhoodoo surfaces	36.0	S56
36	Water	A carbon nanofiber substrate	0.97–1.14	S57
37	Water	A deep–water pool	1.62–1.78	S27
38	Water	Superhydrophobic textured surfaces	1.14–1.59	S58
39	Hot water (52.7 °C)	Superhydrophobic substrate	0.85	S14
40	Water	Hydrophobic rare-earth oxide ceramics	1.19–1.56	S15
41	Water<br>Hexadecane	Candle soot templated superamphiphobic coatings	0.43–1.04	S18
42	Acetic acid<br>Hexylamine<br>PMMA in DMF	Electrospun superomniphobic surfaces	0.92–1.39	S19
43	Water<br>Methanol<br>FC-72	Doubly reentrant structures	0.65–5.78	S20
44	Water<br>Molten tin	Superhydrophobic surfaces with macroscopic ridges and other engineered and natural surfaces	0.81–1.43	S24
45	Silicone oil	A vibrated silicone oil bath	~1.90	S25
46	Hydrocarbon	Hydrocarbon droplet	0.95–1.14	S6
47	Water<br>Soap water	A sessile droplet of the same liquid	0.95–1.07	S28
48	Water	Water droplet on a superhydrophobic surface	1.15	S29
49	Aqueous polyelectrolyte	Superhydrophobic silicon nanograss texture	0.94	S59
50	Water	Water droplet on a superhydrophobic copper plate	1.02–1.03	S3
51	Water	Water bath surface	2.64	S60

52	Hydrocarbon	Hydrocarbon droplet	1.02–1.07	S61
53	Water	Topographically patterned surfaces at 220–310 °C	1.01–1.18	S62
54	Water	Superhydrophobic surfaces with macroscopic strips and other natural leaf surfaces	0.87–1.32	S63
55	Heptane	Hot metallic surface at 180–200 °C	1.07–1.78	S64
56	Tetradecane	Tetradecane droplet	0.89–0.94	S65
57	Propanol	A deep pool of propanol	1.63–2.33	S66
58	Water	Spray-coated superomniphobic surfaces	0.95	S67
59	Tetradecane<br>Dodecane	A shallow layer of the same liquid	1.01–2.11	S68
60	20% Glycerol	A horizontal soap film	1.27–1.91	S69
61	Silicone oil	A vibrating bath of silicone oil	1.27–2.79	S70
62	Silicone oil	A vibrating bath of silicone oil	0.98–3.16	S71
63	Silicone oil	A vibrating bath of silicone oil	~1.23	S72

References

[S1] Deng, T. *et al*. Nonwetting of impinging droplets on textured surfaces. *Appl. Phys. Lett.* **94**, 133109 (2009).

[S2] Ashgriz, N. & Poo, J. Y. Coalescence and separation in binary collisions of liquid drops. *J. Fluid Mech.* **221**, 183–204 (1990).

[S3] Mertaniemi, H., Forchheimer, R., Ikkala, O. & Ras, R. H. Rebounding droplet–droplet collisions on superhydrophobic surfaces: From the phenomenon to droplet logic. *Adv. Mater.* **24**, 5738–5743 (2012).

[S4] Estrade, J.-p., Carentz, H., Lavergne, G., Biscos, Y. Experimental investigation of dynamic binary collision of ethanol droplets – a model for droplet coalescence and bouncing. *Int. J. Heat Fluid Flow* **20**, 486–491 (1999).

[S5] de Ruiter, J., Lagraauw, R., van den Ende, D. & Mugele, F. Wettability-independent bouncing on flat surfaces mediated by thin air films. *Nat. Phys.* **11**, 48–53 (2014).

[S6] Qian, J. & Law, C. K. Regimes of coalescence and separation in droplet collision. *J. Fluid Mech.* **331**, 59–80 (1997).

[S7] Brazier-Smith, P. R., Jennings, S. G. & Latham, J. The interaction of falling water drops: Coalescence. *Proc. R. Soc. A* **326**, 393–408 (1972).

[S8] Pan, S. *et al*. Coatings super-repellent to ultralow surface tension liquids. *Nat. Mater.* **17**, 1040–1047 (2018).

[S9] Clanet, C., Béguin, C., Richard, D. & Quéré, D. Maximal deformation of an impacting drop. *J. Fluid Mech.* **517**, 199–208 (2004).

[S10] Planchette, C., Hinterbichler, H., Liu, M., Bothe, D. & Brenn, G. Colliding drops as coalescing and fragmenting liquid springs. *J. Fluid Mech.* **814**, 277–300 (2017).

[S11] Ching, B., Golay, M. W. & Johnson, T. J. Droplet impacts upon liquid surfaces. *Science* **226**, 535–537 (1984).

[S12] Israelachvili, J. *Intermolecular and Surface Forces* 3rd Edition (Academic Press, 2011).

[S13] Rayleigh, L. On the capillary phenomena of jets. *Proc. R. Soc. London* **29**, 71–79 (1879).

[S14] Shiri, S. & Bird, J. C. Heat exchange between a bouncing drop and a superhydrophobic substrate. *Proc. Natl. Acad. Sci. U. S. A.* **114**, 6930–6935 (2017).

[S15] Azimi, G., Dhiman, R., Kwon, H. M., Paxson, A. T. & Varanasi, K. K. Hydrophobicity of rare-earth oxide ceramics. *Nat. Mater.* **12**, 315–320 (2013).

[S16] Lu, Y. *et al*. Robust self-cleaning surfaces that function when exposed to either air or oil. *Science* **347**, 1132–1135 (2015).

[S17] Richard, D., Clanet, C. & Quéré, D. Contact time of a bouncing drop. *Nature* **417**, 811 (2002).

[S18] Deng, X., Mammen, L., Butt, H.-J. & Vollmer, D. Candle soot as a template for a transparent robust superamphiphobic coating. *Science* **335**, 67–70 (2012).

[S19] Pan, S., Kota, A. K., Mabry, J. M. & Tuteja, A. Superomniphobic surfaces for effective chemical shielding. *J. Am. Chem. Soc.* **135**, 578–581 (2013).

[S20] Liu, T. L. & Kim, C.-J. C. Turning a surface superrepellent even to completely wetting liquids. *Science* **346**, 1096–1100 (2014).

[S21] Liu, Y. *et al*. Pancake bouncing on superhydrophobic surfaces. *Nat. Phys.* **10**, 515–519 (2014).

[S22] Liu, Y., Andrew, M., Li, J., Yeomans, J. M. & Wang, Z. Symmetry breaking in drop bouncing on curved surfaces. *Nat. Commun.* **6**, 10034 (2015).

[S23] Gauthier, A., Symon, S., Clanet, C. & Quéré, D. Water impacting on superhydrophobic macrotextures. *Nat. Commun.* **6**, 8001 (2015).

[S24] Bird, J. C., Dhiman, R., Kwon, H. M. & Varanasi, K. K. Reducing the contact time of a bouncing drop. *Nature* **503**, 385–388 (2013).

[S25] Couder, Y., Protière, S., Fort, E. & Boudaoud, A. Walking and orbiting droplets. *Nature* **437**, 208 (2005).

[S26] Hao, C. *et al*. Superhydrophobic-like tunable droplet bouncing on slippery liquid interfaces. *Nat. Commun.* **6**, 7986 (2015).

[S27] Zou, J., Wang, P. F., Zhang, T. R., Fu, X. & Ruan, X. Experimental study of a drop bouncing on a liquid surface. *Phys. Fluids* **23**, 044101 (2011).

[S28] Wang, F.-C., Feng, J.-T. & Zhao, Y.-P. The head-on colliding process of binary liquid droplets at low velocity: High-speed photography experiments and modeling. *J. Colloid Interface Sci.* **326**, 196–200.

[S29] Yi, N. *et al*. Temperature-induced coalescence of colliding binary droplets on superhydrophobic surface. *Sci. Rep.* **4**, 4303 (2014).

[S30] Li, X., Ma, X. & Lan, Z. Dynamic behavior of the water droplet impact on a textured hydrophobic/superhydrophobic surface: The effect of the remaining liquid film arising on the pillars' tops on the contact time. *Langmuir* **26**, 4831–4838 (2010).

[S31] Lv, C., Hao, P., Zhang, X. & He, F. Drop impact upon superhydrophobic surfaces with regular and hierarchical roughness. *Appl. Phys. Lett.* **108**, 141602 (2016).

[S32] Mundo, R. D., Bottiglione, F., Palumbo, F., Notarnicola, M. & Carbone, G. Filamentary superhydrophobic Teflon surfaces: Moderate apparent contact angle but superior air-retaining properties. *J. Colloid Interface Sci.* **482**, 175–182 (2016).

[S33] Shen, Y., Liu, S., Zhu, C., Tao, J. & Wang, G. Facile fabrication of hierarchical structured superhydrophobic surface and its ultra dynamic water repellency. *Chem. Eng. J.* **313**, 47–55 (2017).

[S34] Wang, J., Liu, M., Ma, R., Wang, Q. & Jiang, L. In situ wetting state transition on micro- and nanostructured surfaces at high temperature. *ACS Appl. Mater. Interfaces* **6**, 15198–15208 (2014).

[S35] Zhang, R., Hao, P. & He, F. Rapid bouncing of high-speed drops on hydrophobic surfaces with microcavities. *Langmuir* **32**, 9967–9974 (2016).

[S36] Lee, W.-K., Jung, W.-B., Nagel, S. R. & Odom, T. W. Stretchable superhydrophobicity from monolithic, three-dimensional hierarchical wrinkles. *Nano Lett.* **16**, 3774–3779 (2016).

[S37] Wong, W. S. Y *et al*. Omnidirectional self-assembly of transparent superoleophobic nanotextures. *ACS Nano* **11**, 587–596 (2017).

[S38] Song, M. *et al*. Controlling liquid splash on superhydrophobic surfaces by a vesicle surfactant. *Sci. Adv.* **3**, e1602188 (2017).

[S39] Nishimura, R. *et al*. Fractal surfaces of molecular crystals mimicking lotus leaf with phototunable double roughness structures. *J. Am. Chem. Soc.* **138**, 10299–10303 (2016).

[S40] Kolinski, J. M., Mahadevan, L. & Rubinstein, S. M. Drops can bounce from perfectly hydrophilic surfaces. *Europhys. Lett.* **108**, 24001 (2014).

[S41] Schutzius, T. M. *et al*. Spontaneous droplet trampolining on rigid superhydrophobic surfaces. *Nature* **527**, 82–85 (2015).

[S42] Vasileiou, T., Gerber, J., Prautzsch, J., Schutzius, T. M. & Poulikakos, D. Superhydrophobicity enhancement through substrate flexibility. *Proc. Natl. Acad. Sci. U. S. A.* **113**, 13307–13312 (2016).

[S43] Weisensee, P. B., Tian, J., Miljkovic, N. & King, W. P. Water droplet impact on elastic superhydrophobic surfaces. *Sci. Rep.* **6**, 30328 (2016).

[S44] Liu, Y. & Wang, Z. Superhydrophobic porous networks for enhanced droplet shedding. *Sci. Rep.* **6**, 33817 (2016).

[S45] Mishchenko, L. *et al*. Design of ice-free nanostructured surfaces based on repulsion of impacting water droplets. *ACS Nano* **4**, 7699–7707 (2010).

[S46] Richard, D. & Quéré, D. Bouncing water drops. *Europhys. Lett.* **50**, 769–775 (2000).

[S47] Bartolo, D. *et al*. Bouncing or sticky droplets: Impalement transitions on superhydrophobic micropatterned surfaces. *Europhys. Lett.* **74**, 299–305 (2006).

[S48] Bartolo, D., Josserand, C. & Bonn, D. Retraction dynamics of aqueous drops upon impact on non-wetting surfaces. *J. Fluid Mech.* **545**, 329–338 (2005).

[S49] Reyssat, M., Richard, D., Clanet, C.& Quéré, D. Dynamical superhydrophobicity. *Faraday Discuss.* **146**, 19–33 (2010).

[S50] Wachters, L. H. J. & Westerling, N. A. J. The heat transfer from a hot wall to impinging water drops in the spheroidal state. *Chem. Eng. Sci.* **21**, 1047–1056 (1966).

[S51] Aziz, S. D. & Chandra, S. Impact, recoil and splashing of molten metal droplets. *Int. J. Heat Mass Transfer* **43**, 2841–2857 (2000).

[S52] Legendre, D., Daniel, C. & Guiraud, P. Experimental study of a drop bouncing on a wall in a liquid. *Phys. Fluids* **17**, 097105 (2005).

[S53] Reyssat, M., Pépin, A., Marty, F., Chen, Y. & Quéré, D. Bouncing transitions on microtextured materials. *Europhys. Lett.* **74**, 306–312 (2006).

[S54] Jung, Y. C. & Bhushan, B. Dynamic effects of bouncing water droplets on superhydrophobic surfaces. *Langmuir* **24**, 6262–6269 (2008).

[S55] Brunet, P., Lapierre, F., Thomy, V., Coffinier, Y. & Boukherroub, R. Extreme resistance of superhydrophobic surfaces to impalement: Reversible electrowetting related to the impacting/bouncing drop test. *Langmuir* **24**, 11203–11208 (2008).

[S56] Tuteja, A., Choi, W., Mabry, J. M., McKinley, G. H. & Cohen, R. E. Robust omniphobic surfaces. *Proc. Natl. Acad. Sci. U. S. A.* **105**, 18200–18205 (2008).

[S57] Tsai, P., Pacheco, S., Pirat, C., Lefferts, L. & Lohse, D. Drop impact upon micro- and nanostructured superhydrophobic surfaces. *Langmuir* **25**, 12293–12298 (2009).

[S58] Kwon, D. H. & Lee, S. J. Impact and wetting behaviors of impinging microdroplets on superhydrophobic textured surfaces. *Appl. Phys. Lett.* **100**, 171601 (2012).

[S59] Damak, M., Mahmoudi, S. R., Hyder, M. N. & Varanasi, K. K. Enhancing droplet deposition through *in-situ* precipitation. *Nat. Commun.* **7**, 12560 (2016).

[S60] Jayaratne, O. W. & Mason, B. J. The coalescence and bouncing of water drops at an air/water interface. *Proc. Royal Soc. A* **280**, 545–565 (1964).

[S61] Jiang, Y. J., Umemura, A. & Law, C. K. An experimental investigation on the collision behaviour of hydrocarbon droplets. *J. Fluid Mech.* **234**, 171–190 (1992).

[S62] Li, J. *et al*. Directional transport of high-temperature Janus droplets mediated by structural topography. *Nat. Phys.* **12**, 606–612 (2016).

[S63] Song, M. *et al*. Reducing the contact time using macro anisotropic superhydrophobic surfaces—effect of parallel wire spacing on the drop impact. *NPG Asia Mater.* **9**, e415 (2017).

[S64] Chandra, S. & Avedisian, C. T. On the collision of a droplet with a solid surface. *Proc. R. Soc. London, Ser. A* **432**, 13–41 (1991).

[S65] Pan, K.-L., Law, C. K. & Zhou, B. Experimental and mechanistic description of merging and bouncing in head-on binary droplet collision. *J. Appl. Phys.* **103**, 064901 (2008).

[S66] Zhao, H., Brunsvold, A. & Munkejord, S. T. Transition between coalescence and bouncing of droplets on a deep liquid pool. *Int. J. Multiphase Flow* **37**, 1109–1119 (2011).

[S67] Vahabi, H., Wang, W., Davies, S., Mabry, J. M. & Kota, A. K. Coalescence-induced self-propulsion of droplets on superomniphobic surfaces. *ACS Appl. Mater. Interfaces* **9**, 29328–29336 (2017).

[S68] Pan, K. L. & Law, C. K. Dynamics of droplet–film collision. *J. Fluid Mech.* **587**, 1–22 (2007).

[S69] Gilet, T. & Bush, J. W. M. The fluid trampoline: Droplets bouncing on a soap film. *J. Fluid Mech.* **625**, 167–203 (2009).

[S70] Moláček, J. & Bush, J. W. M. Drops bouncing on a vibrating bath. *J. Fluid Mech.* **727**, 582–611 (2013).

[S71] Moláček, J. & Bush, J. W. M. Drops walking on a vibrating bath: Towards a hydrodynamic pilot-wave theory. *J. Fluid Mech.* **727**, 612–647 (2013).

[S72] Wind-Willassen, Ø., Moláček, J., Harris, D. M. & Bush, J. W. M. Exotic states of bouncing and walking droplets. *Phys. Fluids* **25**, 082002 (2013).
